# A Scoping Review of Pathogenesis, Current Treatments, and Novel Approaches for Vitiligo

**DOI:** 10.1111/jocd.70444

**Published:** 2025-10-13

**Authors:** Nusaibah Sallehuddin, Nur Izzah Md Fadilah, Mh Busra Fauzi, Manira Maarof

**Affiliations:** ^1^ Department of Tissue Engineering and Regenerative Medicine, Faculty of Medicine Universiti Kebangsaan Malaysia Kuala Lumpur Malaysia; ^2^ Advance Bioactive Materials‐Cells UKM Research Group Universiti Kebangsaan Malaysia Bangi Malaysia; ^3^ Ageing and Degenerative Disease UKM Research Group Universiti Kebangsaan Malaysia Bangi Malaysia

**Keywords:** autoimmunity, melanocyte, repigmentation, stress, vitiligo

## Abstract

**Background:**

Vitiligo is a chronic skin disorder characterized by loss of melanocytes, causing depigmentation and psychological distress. Its pathogenesis is complex and multifactorial, involving genetic predisposition, autoimmune, oxidative stress, and neurogenic mechanisms.

**Aims:**

This scoping review summarizes current understanding of vitiligo pathogenesis and provides an overview of available and emerging treatment modalities.

**Methods:**

A systematic search was conducted in Web of Science, Scopus, and EBSCO Medline for English‐language publications from January 2019 onwards. Eligible studies included those investigating the pathogenesis, therapeutic approaches, or novel strategies for idiopathic vitiligo across all age groups and clinical types. Exclusion criteria included secondary vitiligo due to drugs, comorbidities, or systemic diseases, as well as studies involving pregnant or lactating women and those reporting negative findings.

**Results:**

The search identified 5298 relevant articles. Established therapies include topical and oral agents, phototherapy, surgical interventions, and combination regimens. Recent advances highlight novel therapeutic targets and experimental interventions, many of which are not yet approved but show promising preliminary outcomes. These include immune‐modulating agents, antioxidants, and regenerative strategies aimed at restoring melanocyte function and repigmentation.

**Conclusions:**

Vitiligo remains a challenging dermatological disorder with significant clinical and psychosocial implications. Although existing therapies provide variable outcomes, emerging treatments offer encouraging prospects. This review provides a comprehensive resource for clinicians and researchers, guiding future directions in vitiligo management and therapeutic innovation.

## 1 Introduction

According to Feng and Lu (2022), vitiligo is a circumscribed or generalized skin and mucosal depigmentation characterized by pale patches caused by the selective loss of melanocytes [1]. The distinguishing feature of vitiligo is a totally amelanotic, non‐scaly, pale‐white macule with well‐defined margins [2]. Worldwide, vitiligo affects approximately 0.1%–2% of the population [3]. The pathogenesis of vitiligo includes autoimmune mechanisms in conjunction with genetic and environmental factors, metabolic and oxidative stress, and cell detachment abnormalities [2]. It is crucial not to trivialize or underestimate vitiligo as a mere cosmetic issue, as it can have severe psychological consequences, often leading to stigmatization and social isolation for affected individuals [3]. Vitiligo is categorized into two major forms: nonsegmental vitiligo (NSV) and segmental vitiligo (SV) [2]. This classification holds significance because the treatment approaches differ based on the type and form [2].

Available treatment modalities for vitiligo include phototherapy, surgical intervention, systemic corticosteroids, and topical therapies such as glucocorticoids, immunosuppressants, calcineurin inhibitors, and vitamin C [1]. These therapeutic approaches primarily aim to modulate the immune and inflammatory systems in an unspecified manner. These treatments can be administered individually or in combination to stimulate melanocyte regeneration [3]. However, the current treatment options for vitiligo remain suboptimal and may exhibit varying effectiveness among different vitiligo patients. Additionally, the requirement for clinic visits for phototherapy can pose an inconvenience for patients. Advancements in comprehending the immune‐based pathogenesis of vitiligo have paved the way for the development of targeted therapies. Ongoing clinical trials are exploring the efficacy of biologics that target cytokines and small‐molecule inhibitors that target intracellular signaling molecules. These developments highlight the crucial role played by the immune system in vitiligo [1].

Current cell‐based treatments for vitiligo include autologous melanocyte transplantation, melanocytes‐keratinocytes cell transplantation, the ReCell system, epidermal cell grafting, autologous platelet‐rich plasma, and combination therapy with narrowband ultraviolet B (NB‐UVB) [4]. These cell‐based treatment approaches are continually being improved to achieve better repigmentation outcomes and simplify the methods, thereby increasing their accessibility in dermatological clinics. Procedural modifications focus on simplifying cell collection, ensuring optimal transplantation potential, and utilizing less complex laboratory equipment, which helps reduce the procedure's cost and enhances its availability to patients. The process of repigmentation occurs gradually and may extend beyond 12 months [4]. Thus, there is a need for more extensive follow‐up studies, lasting at least six months, to evaluate the treatment's effectiveness and calculate the actual financial cost that the patients must bear throughout their vitiligo treatment journey.

A scoping review is a form of scientific methodology that addresses an exploratory research question with the aim of mapping key concepts and gaps related to a defined area or field [5]. The updated pathogenesis and treatments for vitiligo are broad research areas. To circumvent the significant resources required to conduct vitiligo‐related investigations, it was deemed essential to review the available literature to date. Therefore, we performed a scoping review to broadly summarize all the available evidence presented to date on the pathogenesis, current treatments, and novel approaches for vitiligo.

## 2 Methodology

The scoping review methodology outlined by the Joanna Briggs Institute [6] was applied, and the results were presented utilizing the recent Preferred Reporting Items for Systematic Reviews and Meta‐Analyses (PRISMA) Extension for Scoping Reviews (PRISMA‐ScR) guidelines [7]. It is important to note that this review article is based on pre‐existing studies; hence, ethical approval from institutional committees was not necessary.

### 2.1 Search Strategy and Study Selection

The aim of this scoping review was to comprehensively summarize the updated pathogenesis, current treatment options, and new approaches pertaining to vitiligo. By mapping the existing literature in this field, the intention was to establish a basis for developing a new approach that would facilitate prompt and complete healing of vitiligo. The keyword used was “vitiligo” The Web of Science, Scopus, and EBSCO Medline databases were selected, and a search was conducted spanning the last 5 years (2019 to 2023). The search was limited to original articles written in English and containing abstracts. Inclusion criteria encompassed studies addressing pathogenesis, treatments, and new approaches to idiopathic vitiligo across all types and age groups. Exclusion criteria were (i) secondary vitiligo, which is vitiligo secondary to drugs such as imiquimod, carbamazepine, or chloroquine, as well as diseases such as rheumatoid arthritis, systemic lupus erythematosus, diabetes, and vaccinations such as COVID‐19 vaccines, (ii) vitiligo with other comorbidities such as infections, metabolic secretory diseases, or other autoimmune diseases, (iii) vitiligo in pregnant and lactating women, (iv) and negative results.

### 2.2 Data Extraction and Analysis

**FIGURE 1:**
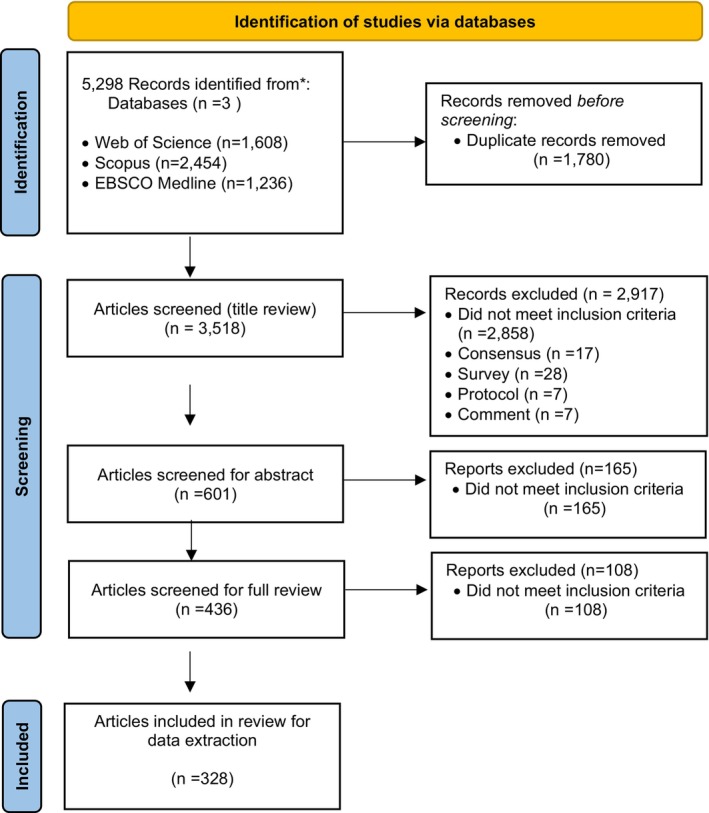
PRISMA flow diagram of illustrating literature search and selection process for inclusion in the systematic review.

The initial screening was based on titles and abstracts and was conducted independently by two reviewers (N.S and M.M). The full texts of potentially eligible studies were then retrieved manually for a more detailed evaluation according to the predefined inclusion and exclusion criteria. The extracted data were tabulated to include updated information on the pathogenesis, current treatment options, and new approaches related to vitiligo. The scope of the new treatment approach encompasses investigations conducted the in vitro, in vivo, and in silico. A cumulative total of 5298 articles pertaining to vitiligo were obtained, with 1608 sourced from Web of Science, 2454 from Scopus, and 1236 from EBSCO Medline. Out of these, 1780 duplicate articles were excluded, and 3083 articles were eliminated after screening the titles and abstracts. Subsequently, the full text of the remaining articles was read, in which 108 articles did not fulfill the inclusion criteria. Thus, 328 articles were finally analyzed in this scoping review, consisting of 312 original studies and 16 case reports (Figure 1).

## 3 Pathogenesis

The exact pathogenesis of vitiligo remains unclear, but several mechanisms have been studied for this noncontagious disease including morphology alteration, DNA damage, abnormal long noncoding RNA, oxidative stress, and autoimmunity. In the early stage of vitiligo, the disease was characterized by progressive degenerative disease with initial premature aging and impaired melanosome transfer [8]. Morphologically, melanocytes with decreased DJ‐1 levels showed altered morphology, including shorter or missing dendrites, decreased cell viability, decreased basal respiration, reduced ATP production, decreased proton leak, and increased apoptosis [9]. The destabilization of melanocytes in the basal layer of the epidermis led to melanocyte loss [10]. Furthermore, DNA and melanophore damage led to activation of the innate immune response and subsequent melanocyte apoptosis [8, 11]. Abnormal long noncoding RNAs influenced melanogenesis‐related genes which were DCT, TYR, and TYRP1 [12]. Proinflammatory factors including lysophosphatidylcholine (LPC), platelet‐activating factor (PAF), sialic acid, CXCL4, and CXCL7 were significantly increased in vitiligo [13]. Activated melanocytes expressed surface molecules and presented their own antigens, triggering T‐cell proliferation and the release of cytokines and chemokines that recruit immune cells to the skin, before their elimination from the basal layer of the epidermis [14, 15].

Increased hydrogen peroxide (H_2_O_2_) level may cause an imbalance in redox homeostasis and trigger the hydroxylation process, affecting substances like tyrosine [16]. High doses of H_2_O_2_ caused cellular damage and death, while low doses induced cell senescence. Premature senescence is considered a significant factor in hypopigmentation [8]. Also, pathological activation of melatonin receptors leads to the uncontrolled production of ROS [17]. Melanocortin 1 receptor (MC1R), a receptor influencing melanocyte development, was found to be downregulated in vitiligo [18]. Oxidative stress induced melanocyte death through apoptosis, autophagy, melanogenesis suppression, and alterations in various signaling pathways [19]. Oxidative stress led to oxeiptosis and release of chemokines that induce the infiltration of CD8+ T cells and maturation of dendritic cells, resulting in autoimmunity [20, 21]. Oxidative stress also impairs dendrite formation in melanocytes and reduces melanosome transfer ability [8]. Increased oxidative factors may facilitate the opening of mitochondrial permeability transition pore [22]. Melanocyte apoptosis led to the formation of apoptotic bodies and the transfer of autoantigens such as tyrosinase, TRP1, TRP2, lamin A, nucleosomes, and histones from melanocytes to these apoptotic bodies, which stimulated autoimmunity [23]. ROCKI plays a crucial role in apoptotic bodies formation [23]. Furthermore, decrease in serum total antioxidant status led to a reduction in neutralizing melanocyte‐damaging oxidant activity [24].

Oxeiptosis was characterized by the dephosphorylation of apoptosis‐inducing factor mitochondria‐associated 1 (AIFM1) at Ser116, regulated by kelch‐like ECH‐associated protein 1 (KEAP1) and phosphoglycerate mutase 5 (PGAM5). KEAP1 functions as a sensor for oxidative stress and degrades nuclear factor erythroid‐2 (Nrf2) under normal conditions [20]. PGAM5 plays a role in mitochondrial dynamics, programmed cell death, and protein phosphatase activity. AIFM1 is a multifunctional cell death effector that is involved in apoptosis and parthanatos (a caspase‐independent PARP‐1‐dependent cell death). AIFM1 is located in the intermembrane space, mitochondria, or outer mitochondrial membrane. In parthanatos, pathologically elongated and branched PAR polymer triggered the release of AIFM1 from the outer mitochondrial membrane, leading to DNA fragmentation. During oxidative stress, KEAP1 dissociated PGAM5 [20]. Multiple affected family members with vitiligo have a higher risk compared to simplex cases. Polygenic risk score is proportional to the number of affected relatives within a family [25]. The pathogeneses of vitiligo are mentioned in Table 1.

**TABLE 1:** Pathogenesis of vitiligo.

Pathogenesis	Study characteristics	References
FOXP3 gene (rs3761548) induced reduction of regulatory T cells (Treg) leading to autoimmune response	Not stated	[26]
IFN‐γ stimulated HDF to secrete CCL2 and CCL8 through JAK/STAT pathway. CCL2 promoted polarization of naïve T cells into Th2 cells, while CCL8 attracted Th2 cells, leading to autoimmune response	Vitiligo mouse model	[27]
CIRP stimulated T cells through NF‐kB proteins, which induced the release of IL‐1B, IL‐8, IL‐6, TNF‐α and HMGB1. CIRP also activated NLRP3 and induced production of IL‐1B	Nonsegmental vitiligo	[28]
Fatty acid‐binding protein 4 (FABP4) upregulated inflammatory cytokines, autoreactive tissue‐resident memory T cells (TRM), endothelial cells, and immune‐mediated process. The modification of membrane lipid components in melanocytes lead to mitochondrial impairment and production of intracellular ROS	Not stated	[29]
Lipid metabolism stimulated vitiligo through PPAR signaling pathway, with leptin and leptin receptor (LEPR) as the representative genes. Leptin enhanced cytotoxic function of CD8+ T cells by affecting cell adhesion molecule	Nonsegmental vitiligo, mouse models of vitiligo	[30]
Oxidative stress stimulated NK cells to secrete IFN‐γ. IFN‐γ triggered the production of CXCL10 through JAK1/2‐STAT1 signaling pathway, which bind CXCR3 on CD8+ T cells and initiated adaptive response. CD8+ cells further upregulated CXCL10 expression and further recruited CD8+ T cells targeting melanocyte destruction. CD8+ T cells migration via the CXCL10/CXCR3 axis was regulated by defective in culin neddylation 1 domain containing 1 (DCUN1D1) gene. Nrf2 signaling activity which is essential to combat oxidative stress, were disordered in vitiligo. CXCL9, CXCL10, and CXCL11 (IFN‐γ dependent chemokines), were found to be involved in IFN‐γ specific Th1 immune responses. High levels of IL‐6, IL‐1α and TNF‐α were found in vitiligo patients. Vitiliginous keratinocytes were susceptible to TNF‐α mediated apoptosis through NF‐κB pathway	Nonsegmental vitiligo [31]	[32], [31]
IFN‐γ, CXCL9, and CXCL10 recruited cytotoxic T cells to the skin	Nonsegmental, generalized vitiligo	[33]
Absence of IFN‐γ induced PD‐L1 expression by melanocytes, suggest that melanocytes cannot inhibit attack (by autoreactive T cells) through PD‐1/PD‐L1 pathway. The presence of programmed cell death protein 1 (PD‐1+) cells correlated positively with cytotoxic T lymphocyte‐associated protein 4 (CTLA‐4) in vitiligo. The author proposed that PD‐1+ cells in vitiligo are mainly melanocyte‐reactive CD8+ resident memory T cells, which have been associated with depigmentation process	Nonsegmental vitiligo	[34]
Low sTAS levels suggesting impairment in neutralizing melanocyte‐damaging oxidant	Nonsegmental vitiligo	[24]
Highly expressed miR‐21‐5p exosomes from peripheral blood suppressed melanogenesis via targeting SATB1		[35]
miR‐125b‐p inhibit melanogenesis by downregulating MITF		[36]
Rs1800482 G>C promoter polymorphism increases iNOS expression and nitric oxide production, leading to oxidative stress and reduced melanocyte adhesion to the extracellular matrix components	Nonsegmental vitiligo	[37]
ADAM10 was reduced while ADAM17 was increased in vitiligo. ADAM17 stimulated the release of TNF‐α and EGFR ligands	Segmental and nonsegmental vitiligo	[38]
Transcription factor HIF‐1α, which is induced by oxidative stress, regulated overexpression of OCLN on CD8+ T cells, which enhances CD8+ T cells migration to the skin. OCLN on CD8+ T cells bound to OCLN on the melanocytes, mediating adhesion of CD8+ T cells to melanocytes, and eventually melanocytes death. After most of melanocytes were destroyed, HDF may participate in maintaining CD8+ T cells by overexpressing OCLN. OCLN may also be involved in maintain TRM in the skin	Nonsegmental vitiligo	[39]
RIP1 (mediator of cell death) forms a necrosome with RIP3, leading to the activation and translocation of p‐MLKL to melanocyte membranes. Mitochondrial ROS promoted the formation of this necrosome	Not stated	[40]
IL‐15 promoted maturation of TRM	Nonsegmental vitiligo	[41]
Treg cannot suppress the increased activity of CD8+ T cells	Active vitiligo	[42]
Overexpression of SFRP5 suppressed melanogenesis through Wnt/β‐catenin signaling pathway	Not stated	[43]
Genetic variations in Nr2 and Keap1 genes, particularly rs35652124 and rs6721961	Not stated	[44]
Downregulation of genes in the calcium signaling pathway and Rap1 signaling pathway impaired function of Treg	Not stated	[45]
Th1 and Th17 cells secreted TNF‐α, IL‐6, and IL‐17 leading to melanocytes apoptosis	Generalized vitiligo	[46]
Dysregulation of zinc synthesis by IL‐17, IL‐4, and IL‐6 cytokines. IL‐17 recruited neutrophils and macrophages to the antigen site and stimulated the production of IL‐1β and TNF‐α. IL‐6 enhanced the expression of intercellular adhesion molecule (ICAM‐1) and inducing polyclonal activation of B‐lymphocytes, leading to melanocytes destruction	Active vitiligo	[47]
Rs1800629 polymorphism was associated with higher TNF‐α levels. Rs1800629 SNP disrupted the SP1 and ATF3 binding site, which could impact the transcription activation by NF‐κB, resulting in increased TNF‐α expression	Not stated	[48]
IFN‐γ inhibited the proliferation of melanocytes and increased their apoptotic rate. IFN‐γ increased the production of p‐JAK1 and p‐STAT1, Bax, Bak, and cleaved caspase‐3, and decreased production of Bcl‐2	Not stated	[49]
Oxidative stress lead to efflux of ATP. Extracellular ATP activated inflammasome in keratinocytes and melanocytes and accumulation of ROS. ATP also induced chemotaxis of CD8+ T cells into the skin by upregulating CXCL9	Active nonsegmental vitiligo	[50]
Reduced expression of NFATC1 and FOXP3 contributed to the dysfunctional Tregs	Active and stable generalized vitiligo	[51]
Zinc‐α2‐glycoprotein (ZAG), which plays a role in melanin production regulation, was decreased in vitiligo	Not stated	[52]
Homocysteine induced apoptosis by activating ROS and endoplasmic reticulum (ER) stress pathway involving protein kinas RNA‐like ER kinase (PERK), eukaryotic translation initiation factor 2α (eIF2α), and C/EBP homologous protein (CHOP). In addition, homocysteine disrupted melanogenesis	Not stated	[53]
Imbalance of Th17/Treg ratio with decreased mRNA levels of Treg specific transcription factors (FOXP3, HELIOS, and EOS) and increased mRNA levels of Th17 specific transcription factor (RORyt)	Active nonsegmental vitiligo	[54]
Accumulation of p16INK4A suggesting intrinsic metabolic defects in melanocytes leading to intracellular oxidative stress	Nonsegmental vitiligo	[55]
Pathological activation of melatonin receptors leads to uncontrolled production of ROS	Stable nonsegmental vitiligo	[17]
Fas receptor interacted with FasL, initiating a cascade leading to apoptosis	Mouse model of vitiligo	[56]
IL‐6 contributed to imbalance of Th17/Treg. IL‐6 interacted with IL6R and induces the transcription of inflammatory genes and reduced expression of MITF and tyrosinase	Not stated	[57]
Prolactin has immunostimulatory effects and promote autoimmunity by influencing T lymphocytes and cytokine production	Vitiligo vulgaris	[58]
Neuropeptides like substance P (SP) stimulated leukocyte activation and cytokine development. The gene for angiotensin‐converting enzyme (ACE) (which can degrade SP) was polymorphed	Not stated	[59]
High saturated fatty acid diet can cause autoimmunity and effect membrane fluidity	Generalized or localized vitiligo	[60]
Increased serum malondialdehyde (MDA) levels destroys melanocytes	Active and stable vitiligo	[61]
Autoantibodies to melanocytes (AMA) targeted antigens in the cytoplasm and membrane, leading to complement‐dependent cytotoxicity (CDC) and antibody‐dependent cell‐mediated cytotoxicity (ADCC), as well as enhancing antigen uptake and presentation by dendritic cells	Nonsegmental vitiligo	[62]
IL‐27 function as both a proinflammatory and anti‐inflammatory mediator	Generalized and localized vitiligo	[63]
TRM expressed CD122 subunit of the IL‐15 receptor, leading to activation of melanocyte destruction	Nonsegmental vitiligo	[64]
Circulating follicular helper T cells (Tfh) stimulated B cells to produce autoantibodies	Nonsegmental vitiligo	[65]
HSP90 triggered oxidative stress leading to immune system activation	Not stated	[66]
FABP4 induced inflammatory responses through NF‐κB and TNF‐α. FABP4 also might influence dendritic cells, endothelial cells, and T‐cell activity. Clusterin secreted by endothelial cells can inhibit melanogenesis	Nonsegmental vitiligo	[67]
Melanocortin 1 receptor (MC1R): A receptor influencing melanocyte development and function was found lower in vitiligo	Not stated	[18]
High expression of HLA‐DR activated Langerhans cells and dendritic cells. Inflammatory dendritic cells stimulated Th17 cells proliferation via IL‐23, triggering cytotoxic T cells to destroy melanocytes	Nonsegmental vitiligo	[68]
Elevated hydrogen peroxide (H_2_O_2_) levels may cause imbalance in redox homeostasis and triggers hydroxylation process, affecting substances like tyrosine	Not stated	[16]
TNF‐α induced cellular and mitochondrial ROS generation, decreased MITF‐M expression, and increased ICAM1, TNFR1, and IL‐6 levels	Active and stable vitiligo	[69]
Low miR‐200 levels	Not stated	[70]
Melanocyte apoptosis mediated by CD8+ T cells and NK cells were mediated by two pathways; perforin/granzyme and Fas/FasL	Active nonsegmental vitiligo	[71]
STAT3 gene variant (rs744166 T>C) intensified the attraction of NK cells to the skin by increasing transcription factor expression. Activated STAT3 increased Th17 cytokine expression	Not stated	[72]
Has_circRNA_000957 and has_circRNA_101798, circRNAs which associated with autoimmunity, were found to be upregulated and downregulated in vitiligo	Not stated	[73]
Hypomethylation of specific CpG sites was found in CD8+ T cells, correlated with overexpression of perforin. HIF‐1α enhanced cytotoxicity by promoting glycolysis and perforin secretion	Active vitiligo	[74]
Increased frequency of Th9 cells in vitiligo	Not stated	[75]
Dysregulated circRNAs were found to target genes associated with melanogenesis. While, dysregulated miRNAs influenced pathways linked to melanogenesis	Vitiligo vulgaris	[76]
Increased inflammation could contribute to elevated epinephrine, norepinephrine, and dopamine, oxidative stress, and melanocyte death	Active vitiligo	[77]
Langerhan cells in vitiligo displayed alteration in morphology	Nonsegmental vitiligo	[78]
Upregulation of p53 combined with downregulation of Bcl‐2 suggests that melanocytes are more susceptible to apoptosis due to the loss of Bcl‐2's protective mechanism	Not stated	[79]
TLR4 gene polymorphisms specifically rs11536858, rs1927914, and rs1927911 modulate autoimmune responses toward vitiligo	Not stated	[80]
Decrease AhR expression on CD4+ T cells lead to increase IL‐17A expression which actives autoimmunity	Unstable vitiligo	[81]
IL‐1 bound to IL‐IR1 and IL‐1R2 receptors, inhibiting melanocyte growth	Not stated	[82]
miR‐21 overexpression influenced SOX5 and MITF, affecting melanogenesis	Nonsegmental vitiligo	[83]
Endoplasmic reticulum (ER) stress‐stimulated autoimmunity. IRE1/XBP1 influenced differentiation of Th17 cells, producing IL‐17A	Not stated	[84]
Oxidative stress leads to IL‐15 expression through activation of NF‐κB signaling. IL‐15 activated unfolded protein response (UPR) in melanocytes and cytotoxic proteins in CD8+ T cells via JAK/STAT pathway	Nonsegmental vitiligo	[85]
Oxidative stress lead to calcium influx and mitochondrial ROS accumulation, activating the NLRP3 inflammasome. IL‐1β/IL‐1R signaling via the NLRP3 inflammasome increased expression of CXCR6 and CXCR3 on CD8+ T cells and augmented production of IL‐17A/F in CD4+ T cells and IFN‐γ in both CD8+ and CD4+ T cells. TRPM2, known for mitochondria‐dependent apoptosis of melanocytes, is implicated in this pathway. IL‐1β promoted the expression of CXCL10 and CXCL16, contributing to the CD8+ T cells infiltration	Active and stable vitiligo	[86]
Oxidative stress increased miR‐421 and ER stress‐related proteins; PERK, eIF2α, and CHOP, also decreased RIPK1, leading to inhibition of melanogenesis and apoptosis	Not stated	[87]
Oxidative stress activated JNK and MAPK, initiating cytochrome c‐mediated caspase pathway resulting in apoptosis. Apoptosis lead to formation of apoptotic bodies (ABs) and transfer of autoantigens such as tyrosinase, TRP1, TRP2, Lamin A, nucleosomes, and histones from melanocyte to these ABs, stimulating autoimmunity. RhoGTPase and ROCKI play a crucial role in ABs formation	Not stated	[23]
Upregulated Th1, Tc1, Th17, and Tc17 cells including CCR6‐expressing T cells in vitiligo	Not stated	[88]
The expression of NKG2D ligands MICA‐MICB activated NKG2D‐expressing CD8+ T cells, reinforced by type I IFN, lead to the production of IFN‐γ and TNF‐α	Not stated	[89]
mRNAs regulated by abnormally expressed lncRNAs direct or indirectly act on melanogenesis‐related genes include DCT, TYR, and TYRP1 to influence melanogenesis	Not stated	[12]
Downregulated LncRNA TUG1 inhibited melanogenesis by affecting the phosphorylation of the ERK/MAPK signaling pathway, which reduced MITF. Upregulated MiRNA‐377 influenced melanocytes indirectly through the secretion of cytokines and growth factors. Decrease PPAR‐c leading to reduction in melanogenesis and antioxidant defense	Not stated	[90]
Acetylcholinesterase (AChE) and nicotinic acetylcholine receptors (nAChRs) is lower in vitiligo while muscarinic acetylcholine receptors (mAChRs) is high.	Nonsegmental vitiligo	[91]
Oxidative stress lead to oxeiptosis, characterized by dephosphorylation of AIFM1 at Ser116, regulated by KEAP1 and PGAM5, resulting in apoptosis. Nrf2 accumulated and translocated to the nucleus, leading to the expression of antioxidant response gene. Under severe mitochondrial damage, PGAM5 lead to excessive mitochondrial fission, abnormal mitochondrial movement, and apoptosis or necroptosis. PGAM5's Ser protein phosphatase activity mediated the dephosphorylation of AIFM1 in melanocytes exposed to oxidative stress. AIFM1 is involved in both apoptosis and parthanatos (a caspase‐independent PARP‐1‐dependent cell death). In parthanatos, pathologically elongated and branched PAR polymer triggers the release of AIFM1 from the outer mitochondrial membrane, leading to DNA fragmentation	Not stated	[20]
The destabilization of melanocytes in the basal layer of the epidermis, driven by E‐cadherin cleavage and active MMP‐9, resulted in melanocyte loss. The upregulation of MMP‐9 and E‐cadherin cleavage was triggered by the IFN‐γ, TNF‐α, IL‐1β, IL‐17, and IL‐6	Not stated	[10]
The combination of Th1/Th2 cytokines enhanced the expression of chemokine ligands which recruited CD8+ T cells. This was regulated by CCL5, CCL18, and CXCL10. CCR5 and CCR8 on these T cells respond to CCL5 and CCL18, respectively, facilitating their migration and activation in the skin	Not stated	) [15]
IFN‐γ and IL‐17A suppressed GPNMB expression in keratinocytes, leading to apoptosis	Not stated	[92]
Reduced plasma calcium and ORAI1 transcripts lead to calcium uptake defects in Treg, resulting in reduction of calcineurin and NFATc1 activation, and subsequent decreased Treg immunosuppressive capacity. Elevated GSK‐3β activity and GSKB and DYRK1A transcripts are involved in reduced NFATc1 activity, resulting in Treg impairment	Not stated	[93]
miRNA‐183‐5p is a direct regulator of MITF in iMC23 melanocytes	Not stated	[94]
The death of melanophores is induced by tyrosinase‐dependent pathways. Damaged melanophores in nicastrin mutants activate an innate immune response	Zebrafish	[11]
Melanocytes with reduced DJ‐1 levels showed shorter or missing dendrites, decreased cell viability, decreased mitochondrial membrane potential, decreased basal respiration, reduced ATP production, decrease proton leak, and increased apoptosis	Not stated	[9]
Overexpression of miR‐493‐3p lead to increased dopamine concentration and ROS, and decrease in melanogenesis by targeting HNRNPU	Segmental vitiligo	[95]
High doses of H_2_O_2_ lead to p21 downregulation and apoptosis, while lower dose caused cell senescence. ROS acts as upstream mediators for MAPK pathway activation, contributing to premature senescence. Oxidative stress impaired dendrite formation and reduced melanosome transfer ability	Not stated	[8]
IFN‐γ reduced 5‐HT‐induced melanogenesis, tryptophan hydroxylase 1 (TPH1), MITFa, and TYRP1a expression. The downregulation of 5‐Ht1A, 5‐HT1B, and 5‐HT2A by IFN‐γ was mediated through IFNGR1 and IFNGR2	Zebrafish	[96]
The downregulation of TRP1 is hypothesized to play a role in melanosome transfer from melanocytes to keratinocytes or in melanosome maturation within keratinocytes	Not stated	[97]
Oxidative stress stimulated NK and ILC1 cells resulting in IFN‐γ production and CXCL such as CXCL10 secretion leading to apoptosis. NK and ILC cells were sensitive to DAMPs, such as HSP70 and HMGB1. CXCR3B expression is elevated in vitiligo patients	Stable vitiligo	[14]
Oxidative stress stimulated HMGB1 secretion and triggered chemokines production. HMGB1 accelerated the maturation of dendritic cells, activating cytotoxic T cells. HSP70 induced an inflammatory response in dendritic cells. Extracellular HMGB1 secreted upon cell death, promoted inflammation by interacting with Pattern Recognition Receptors (PRR), especially receptor for advanced glycation end products (RAGE). Oxidative stress also stimulated the translocation of calreticulin from the ER to the melanocyte's membrane	Active and stable vitiligo	[21]
Proinflammatory factors, including lysophosphatidylcholine (LPC), platelet‐activating‐factor (PAF), sialic acid, CXCL4, and CXCL7, were significantly elevated in vitiligo, inducing the expression of COX2	Not stated	[13]
Oxidative stress inhibited ATP synthase by promoting overexpression of IF1. The increased oxidative factors may facilitate the opening of mitochondrial permeability transition pore, leading to apoptosis	Not stated	[22]
Multiple affected family members with vitiligo have a higher risk compared to simplex cases. Polygenic risk score is proportional to the number of affected relatives within a family. Specific genetic variant related to the MHC class II locus is overrepresented in multiplex‐affected probands	Generalized vitiligo	[25]
Peripheral follicular helper CD4+ T cells help B‐cell function, such as Tfh type 2 and type 17 which produce Th2‐ and Th17‐ related cytokines, respectively	Not stated	[98]
Oxidative stress induced melanocyte death through apoptosis, autophagy, disruption of melanogenesis, and alterations in various signaling pathways	Not stated	[19]
Overexpression of BAFF activated autoreactive B cells and increased the production of autoantibodies against melanocytes. These autoantibodies may activate CD4+ T cells, further exacerbating the immune response	Nonsegmental vitiligo	[99]
Altered expression of ferroptosis markers, iron overload, accumulation of lipid peroxide, and inhibition of melanin synthesis in melanocytes were induced by erastin, which was attenuated by N‐acetyl‐L‐cysteine (NAC)	Not stated	[100]
Substance P (SP) decreased mRNA expression of HPA axis‐related elements and receptors. SP directly activated NK1R on keratinocytes, reducing melanin production	Mice model of vitiligo	[101]
IL‐17A was increased and TGF‐β1 was decreased in vitiligo	Generalized vitiligo	[102]
Thyroid dysfunction rate was low in vitiligo. Personal autoimmune history was inversely associated with elevated IgE	Not stated	[103]
Upregulated TLR4 gene expression led to inhibition of melanogenesis, downregulation of melanin synthesis‐related proteins, and activation of autophagy. LPS inhibited skin pigmentation by modulating autophagy	Not stated	[104]
Elevated GSTT1, GSTA1, and GSTP1 in vitiligo may represent a response to excess free radical formation in vitiligo	Not stated	[105]
Increased serum level of IL‐17 and SAA in vitiligo with positive relation to vitiligo severity and duration of the disease	Not stated	[106]
Tyrosinase, a protein crucial for melanin synthesis, is one of the antigens responsible for the production of antibodies in vitiligo	Not stated	[107]
Macrophage Migration Inhibitory Factor (MIF) activated the proliferation of T cells	Not stated	[108]

### 3.1 Cells Involved in Vitiligo

#### 3.1.1 Regulatory T Cells

Regulatory T cells (Treg) specific transcription factors were FOXP3, HELIOS, and EOS [54]. The reduction of Treg specific transcription factors led to dysfunctional Treg which cannot suppress the increased activity of CD8+ T and CD4+ T cells [26, 42, 51]. Reduction of NFATC1 also induced dysfunctional Treg [51]. Furthermore, reduced plasma calcium and ORAI1 transcripts lead to reduced calcium uptake in Treg, resulting in reduced calcineurin and NFATc1 activation, leading to impaired Treg immunosuppressive functions [45, 93]. In addition, downregulation of genes in the Rap1 signaling pathway impaired the function of Treg [45].

#### 3.1.2 Dendritic Cells

Increased inflammation could contribute to elevated epinephrine, norepinephrine, and dopamine, leading to oxidative stress [77]. Inflammatory dendritic cells which migrated from the skin stimulated Th17 cell proliferation via IL‐23 and presented melanocyte antigens to T cells, resulting in activation of cytotoxic T cells that destruct melanocytes [77].

#### 3.1.3 Resident Memory T Cells

Resident memory T cells (TRM) expressed the CD122 subunit of the IL‐15 receptor, leading to the activation of melanocyte apoptosis [64]. CCL5, CCL18, and CXCL10 were important in recruiting TRM to the skin [15]. CCR5 and CCR8 receptors on TRM respond to CCL5 and CCL18, respectively, facilitating their migration and activation in vitiligo skin [15].

#### 3.1.4 Follicular Helper T Cells

Circulating follicular helper T cells (TFH) stimulated B cells such as Tfh type 2 and 17, producing cytokines and autoantibodies [65, 98].

#### 3.1.5 Cytotoxic T Lymphocytes

Cytotoxic T lymphocytes including CD4+ and CD8+ T cells destruct melanocytes. The presence of CD8+ T cells has been associated with the depigmentation process [34].

#### 3.1.6 Natural Killer Cells

Natural killer (NK) cells and innate lymphoid cells (ILC) were more sensitive to DAMPs, such as HSP70 and HMGB1 [14]. Oxidative stress stimulated NK cells to secrete IFN‐γ [93]. Increased expression of natural killer group 2D (NKG2D) ligand MICA/MICB regulated by type I IFN on the skin activated NKG2D‐expressing CD8+ T cells, which were specific for melanocyte antigens, producing IFN‐γ and TNF‐α [89].

#### 3.1.7 Helper T Cells

Helper T cells (Th)1 and Th17 secrete pro‐inflammatory cytokines, which are TNF‐α, IL‐6, and IL‐17, which led to melanocyte destruction [46]. Imbalance of Th17/Treg ratio with increased mRNA levels of Th17 specific transcription factor (RORyt) leads to the development of vitiligo. Th1, Tc1, Th9, and Th17 cells, including CCR‐6 expressing T cells, were upregulated in vitiligo [75, 88]. The combination of Th1 and Th2 cytokines enhanced the expression of chemokines by melanocytes, which were crucial for recruiting CD8+ T cells into the skin [15]. NK and ILC1 cells were associated with the Th1 immune response [14].

#### 3.1.8 Langerhans Cells

Langerhans cell‐mediated cellular immunity contributed to oxidative stress. In vitiligo, Langerhans cells displayed changes in morphology [78].

#### 3.1.9 Endothelial Cells

Endothelial cells secreted clusterin, which inhibits melanogenesis [67].

### 3.2 Proteins

#### 3.2.1 Ligand

TNF‐related apoptosis‐inducing ligand (TRAIL), a protein functioning as a ligand that induces apoptosis, may also contribute to vitiligo [56].

#### 3.2.2 Hormone

Prolactin has an immunostimulatory effect and promotes autoimmunity by influencing T cells and cytokine production. Prolactin is known to be a neuroendocrine mediator of stress responses [58]. Leptin enhanced the cytotoxic function of CD8+ T cells by affecting cell adhesion molecules [30]. The modification of membrane lipid components in melanocytes may lead to mitochondrial impairment and subsequent production of intracellular ROS [29].

#### 3.2.3 Neuropeptide

Substance P (SP) triggered inflammatory reactions, including leukocyte activation and cytokine development [59]. SP directly activated NK1R on melanocytes' membrane, reducing melanin production. Keratinocytes in the skin express NK1R, and SP indirectly inhibits melanogenesis by affecting keratinocytes [101].

#### 3.2.4 Enzymes

Acetylcholinesterase (Ache) and nicotinic acetylcholine receptors (nAChRs) were downregulated while muscarinic acetylcholine receptors (mAChRs) were upregulated [91]. Angiotensin‐converting enzyme (ACE) can degrade SP. ACE gene polymorphism (D allele, DD genotype, recessive mode) is associated with vitiligo [59]. Rs1800482 G>C promoter polymorphism led to increased inducible nitric oxide synthase (iNOS) expression and nitric oxide production, and subsequently, oxidative stress [37]. Rs1800629 polymorphism was associated with higher TNF‐α levels. Rs1800629 SNP disrupted the SP1 and ATF3 binding sites, which could impact transcription activation by NF‐κB, resulting in increased TNF‐α expression [48]. ATP synthase functions for oxidative phosphorylation and the control pathway related to oxidation and apoptosis. Increased oxidative stress led to inhibition of ATP synthase through overexpression of IF1 [22].

#### 3.2.5 Autoantibodies to Melanocytes

Autoantibodies to melanocytes (AMA) include tyrosinase hydroxylase, tyrosinase, C‐kit, lysosomal‐associated membrane protein‐2, vit‐40, vit‐75, and vit‐90. AMA target antigens in the cytoplasm and cell membrane, contributing to complement‐dependent cytotoxicity (CDC), antibody‐dependent cell‐mediated cytotoxicity (ADCC), and increased antigen uptake and presentation by dendritic cells [62].

#### 3.2.6 Transcription Factor

Signal transducer and activator of transcription 3 (STAT3) gene variant (rs744166 T>C) induced the migration of NK cells to the skin by increasing transcription factor expression [72]. STAT3 protein belongs to the JAK/STAT pathway. Activated STAT3 increased Th17 expression, stimulating fibroblasts and keratinocytes to secrete chemokines that attract neutrophils and NK cells [72].

#### 3.2.7 Circular RNA

Dysregulated circular RNAs (circRNA) were found to target genes associated with melanogenesis [76]. Has_circRNA_000957 and has_circRNA_101798 were found to be upregulated and downregulated in vitiligo. These circRNAs were located on chromosomes associated with autoimmunity, targeting genes associated with vitiligo development [73].

#### 3.2.8 Tumor Suppressor

p53 induced apoptosis [79]. Bcl‐2 is expressed by melanocytes. Upregulation of p53 combined with downregulation of Bcl‐2 suggests that melanocytes are more susceptible to apoptosis due to the loss of Bcl‐2's protective mechanism [79]. p16INK4a is a tumor‐suppressor protein and cyclin‐dependent kinase (cdk) inhibitor that blocks cdk4 and cdk6 mediated pRb phosphorylation to inhibit E2F‐dependent transcription and cell‐cycle progression [109]. The accumulation of p16INK4A suggests intrinsic metabolic defects in melanocytes leading to intracellular oxidative stress [55].

#### 3.2.9 Receptor

Toll‐like receptor 4 (TLR4) gene polymorphism specifically rs11536858, rs1927914, and rs1927911 modulate autoimmunity [26]. TLR4 gene expression was increased in vitiligo [104]. TLR4 ligand lipopolysaccharide inhibits melanogenesis, downregulates the expression of melanin synthesis‐related proteins, and activates autophagy in vitiligo melanocytes [104]. Mitochondrial ROS promotes the formation of receptor‐interacting protein RIP1/RIP3 necrosome, leading to the activation and translocation of p‐MLKL to melanocyte membranes [40].

#### 3.2.10 Damage Associated Molecular Pattern

Damage associated molecular pattern (DAMP) molecules including high mobility group box 1 (HMGB1) were involved in vitiligo. HMGB1 directly induced melanocyte apoptosis. Under oxidative stress, HMGB1 secreted by melanocytes can stimulate the secretion of chemokines from keratinocytes. HMGB1 was secreted into extracellular space upon cell death. This extracellular HMGB1 then promotes inflammation by interacting with Pattern Recognition Receptors (PRR). The predominant PRR that mediates the proinflammatory effects of HMGB1 in keratinocytes is the receptor for advanced glycation end products (RAGE). HMGB1 accelerates the maturation of dendritic cells, which activates cytotoxic T cells targeting melanocytes. HSP70 secreted by melanocytes induced an inflammatory response in dendritic cells [21]. HSP70i triggered TLR4 [80]. HSP90 triggered oxidative stress, leading to immune system activation [66].

#### 3.2.11 Cold‐Inducible RNA‐Binding Protein

Cold‐inducible RNA‐binding protein (CIRP) stimulated T cells through NF‐κB proteins, which induced the release of IL‐1B, IL‐8, IL‐6, TNF‐α, and HMGB1 [28]. CIRP also activated NLRP3 and induced the production of IL‐1B.

#### 3.2.12 Apolipoproteins

Serum amyloid A was increased in vitiligo with a positive relation to the severity and duration of the disease [106].

#### 3.2.13 Antioxidants

Keap1 antioxidants play a crucial role against oxidative stress. Keap1 gene genetic variation, particularly rs6721961, was associated with vitiligo [44]. Malondialdehyde (MDA) is a marker for lipid peroxidation, which reflects the occurrence of oxidative stress. Increased serum MDA levels destroy melanocytes [60]. Peroxisome‐proliferator‐activated receptors‐c, which are involved in melanogenesis and antioxidant defense in melanocytes, were reduced in vitiligo [90].

#### 3.2.14 Organic Compound

Oxidative stress leads to the efflux of adenosine triphosphate (ATP) from melanocytes. Extracellular ATP serves as a primary activator of the inflammasome, mainly controlled by caspase‐1, leading to inflammasome activation in melanocytes and the accumulation of ROS. Moreover, ATP induces chemotaxis of CD8+ T cells into the skin by increasing CXCL9 [50].

#### 3.2.15 Amino Acids

Increased homocysteine levels may play roles in the development of vitiligo [67]. Homocysteine induced apoptosis by activating ROS and endoplasmic reticulum (ER) stress pathway involving protein kinase RNA‐like ER kinase (PERK), eukaryotic translation initiation factor 2α (eIF2α), and C/EBP homologous protein (CHOP) [53]. In addition, homocysteine disrupted melanogenesis [53].

#### 3.2.16 Small Molecule

Erastin reduced cell viability, altered the expression of ferroptosis markers, and inhibited melanogenesis, attenuated by N‐acetyl‐L‐cysteine (NAC) [100].

#### 3.2.17 MicroRNA

Upregulated mmiR‐21‐5p exosomes suppress melanogenesis by targeting SATB1 [35]. miR‐125b‐p inhibited melanogenesis by downregulating MITF, a master transcriptional regulator of melanocyte lineage and melanin synthesis enzymes [36]. miRNA‐183‐5p is a direct regulator of MITF in iMC23 melanocytes [94]. Upregulated miR‐21 can influence SOX5 and MITF, affecting melanogenesis [83]. Meanwhile, upregulated miRNA‐377 was associated with fibroblast dysfunction and may influence melanogenesis [90]. In addition, increased miR‐493‐3p expression led to increased dopamine concentration, increased ROS, melanocyte apoptosis, and a decrease in melanogenesis by targeting HNRNPU [95]. miR‐200 stimulated melanin synthesis by upregulating MITF, TYR, TRP1, TRP2, and β‐catenin. miR‐200 was found to be downregulated in vitiligo [70].

#### 3.2.18 Chemical Element

Zinc is a cofactor for tyrosinase. Zinc is essential in the terminal stage of melanin synthesis. Zinc‐α2‐glycoprotein (ZAG), which plays a role in melanin production regulation, was decreased in vitiligo [52].

### 3.3 Genes

#### 3.3.1 Fatty Acid‐Binding Protein 4

Fatty acid‐binding protein 4 (FABP4) induced the release of inflammatory cytokines through NF‐κB and TNF‐α [67]. FABP4 also stimulated endothelial cells, dendritic cells, and TRM, leading to an autoimmunity response [29, 67]. The resulting inflammatory dendritic cells migrated from the skin, presenting melanocyte antigens to T cells and stimulating autoreactive T cells that destroy melanocytes [67]. FABP4 might contribute to vitiligo progression through dyslipidemia and hyperglycemia [67]. A high‐saturated fatty acid diet can cause autoimmunity and affect membrane fluidity [60].

#### 3.3.2 NALP1

Increased NALP1, which encodes for NACHT leucine‐rich‐repeat protein 1, a regulator of the innate immune system, suggests a possible role in melanocyte apoptosis [54].

#### 3.3.3 Human Leukocyte Antigen—DR Isotype

Overexpressed human leukocyte antigen‐DR isotype (HLA‐DR) activated Langerhans cells and dendritic cells, leading to interaction with melanocytes [68].

#### 3.3.4 Tumor Necrosis Factor Receptor 1

Tumor necrosis factor receptor 1 (TNFR1) induced NF‐κB activation and melanocyte apoptosis [69].

#### 3.3.5 Aryl Hydrocarbon

Decreased aryl hydrocarbon (AhR) expression on CD4+ T cells leads to increased IL‐17A expression, which activates autoimmunity [81].

#### 3.3.6 Glutathione S‐Transferase

Elevated glutathione s‐transferase theta 1 (GSTT1), glutathione s‐transferase alpha 1 (GSTA1), and glutathione s‐transferase pi 1 (GSTP1) in vitiligo may represent a response to excess free radical formation in vitiligo [105].

#### 3.3.7 Intercellular Adhesion Molecule 1

Intercellular adhesion molecule 1 (ICAM1) influenced T‐cell adhesion to melanocytes [69].

#### 3.3.8 Hypoxia‐Inducible Factor 1‐Alpha

Hypoxia‐inducible factor 1‐alpha (HIF‐1α) upregulated the expression of OCLN on CD8+ T cells, which enhanced CD8+ T cell migration to the skin. OCLN on CD8+ T cells bound to OCLN on melanocytes, mediating their adhesion and eventually melanocyte apoptosis. After most melanocytes were destroyed, HDFs may overexpress OCLN to maintain CD8+ T cells. Furthermore, OCLN may also be involved in maintaining TRM in the skin [39]. HIF‐1α enhanced cytotoxicity by promoting glycolysis and perforin secretion. HIF‐1α also might lead to perforin gene promoter hypomethylation by affecting DNMT1 expression [74].

#### 3.3.9 A Disintegrin and Metalloproteinase Domain‐Containing Protein

A disintegrin and metalloproteinase domain‐containing protein (ADAM)10 which functions to degrade cytokines and chemoattractant, was reduced in vitiligo. ADAM17 stimulated the release of TNF‐α and EGFR ligands [38].

#### 3.3.10 Secreted Frizzled Related Protein 5

Upregulated secreted frizzed related protein 5 (SFRP5) suppressed melanogenesis through the Wnt/β‐catenin pathway [43].

#### 3.3.11 Tyrosinase Related Protein 1

The downregulation of tyrosinase related protein 1 (TRP1) plays a role in melanosome transfer from melanocytes to keratinocytes, or melanosome maturation within keratinocytes [97].

### 3.4 Interleukins

#### 3.4.1 IL‐1

High levels of IL‐1α were found in vitiligo patients [31]. IL‐1α inhibits melanocyte growth [82].

#### 3.4.2 IL‐2

IL‐2 exonic variant rs2069763 leads to alterations in protein levels, potentially contributing to vitiligo emergence [66].

#### 3.4.3 IL‐4

IL‐4 dysregulated zinc synthesis [57].

#### 3.4.4 IL‐6

High levels of IL‐6 were found in vitiligo patients [31]. IL‐6 interacts with its receptor IL6R and induces the transcription of inflammatory genes, reduces expression of MITF and tyrosinase, and decreases melanogenesis [57]. IL‐6 enhanced the expression of intercellular adhesion molecule (ICAM‐1) and induced polyclonal activation of B‐lymphocytes, leading to melanocyte destruction [47]. In addition, IL‐6 enriched the microenvironment with proinflammatory signals and contributed to the imbalance of Th17/Treg [57, 69]. Furthermore, IL‐6 dysregulated zinc synthesis [57].

#### 3.4.5 IL‐10

Decrease in IL‐10 resulted in abnormal DNA methylation and depigmentation [68].

#### 3.4.6 IL‐15

Oxidative stress led to IL‐15 expression through the activation of the NF‐κB signaling pathway [85]. IL‐15 expression on keratinocytes activates CD8+ T cells via the JAK/STAT pathway. IL‐15 promoted the maturation of TRM, contributing to the autoimmunity against melanocytes [41].

#### 3.4.7 IL‐17

IL‐17 recruited neutrophils and macrophages, including T cells, stimulating the secretion of IL‐1β and TNF‐α, which induced melanocyte apoptosis [47, 90]. Increased serum level of IL‐17 was related to the severity and duration of vitiligo [106]. Furthermore, IL‐17 dysregulated zinc synthesis [57]. IL‐17A's presence in the skin is associated with the infiltration of Th17 cells and influences keratinocytes and fibroblasts to secrete proinflammatory cytokines, affecting melanocyte homeostasis and melanogenesis [38]. IL‐17A was increased, and TGF‐β1 was decreased in vitiligo [102].

#### 3.4.8 IL‐27

IL‐27 can function as both a proinflammatory and anti‐inflammatory mediator [63].

### 3.5 Cytokines

An imbalance of epidermal cytokines suggests an autoimmune basis for vitiligo [57].

#### 3.5.1 Migration Inhibitory Factor

Macrophage migration inhibitory factor (MIF) activated T cells [108].

#### 3.5.2 Interferon Gamma

Interferon gamma (IFN‐γ) inhibited the proliferation of melanocytes in a time‐dependent manner and increased their apoptotic rate [49]. IFN‐γ stimulated fibroblasts to secrete CCL2 and CCL8 through the JAK/STAT pathway. CCL2 promoted the polarization of naïve T cells into Th2 cells, while CCL8 attracted Th2 cells, leading to an autoimmune response [27]. In addition, IFN‐γ triggered the production of CXCL10 through the JAK1/2‐STAT1 signaling pathway, which binds CXCR3 on CD8+ T cells [32]. CD8+ T cells further increased CXCL10 expression and further recruited CD8+ T cells targeting melanocyte destruction. CD8+ T cell migration via the CXCL10/CXCR axis was regulated by defective culin neddylation 1 domain containing 1 (DCUN1D1) gene. An increase in IFN‐γ leads to an increase in CD4+ and CD8+ cells [28]. IFN‐γ dependent chemokines, which were CXCL9, CXCL10, and CXCL11, were found to be involved in Th1 immune responses [31], recruiting cytotoxic T lymphocytes leading to melanocyte apoptosis [33]. The absence of IFN‐γ induced PD‐L1 expression by melanocytes suggests that melanocytes cannot inhibit attack by autoreactive T cells through the PD‐1/PD‐L1 pathway [34]. IFN‐γ increased the production of p‐JAK1 and p‐STAT1, Bax, Bak, and cleaved caspase‐3, and decreased the production of Bcl‐2 [49]. Glycoprotein non‐metastatic melanoma protein B (GPNMB) is a melanocyte marker crucial for melanosome formation. IFN‐γ and IL‐17A can suppress GPNMB expression in keratinocytes [92]. IFN‐γ reduced 5‐HT, MITFa, TPH1, and TYRP1a. TPH1 is a key enzyme in peripheral 5‐HT synthesis. IFN‐γ reduced the expression of 5‐HT1A, 5‐HT1B, and 5‐HT2A through IFNGR1 and IFNGR2 [96].

#### 3.5.3 Tumor Necrosis Factor Alpha

Tumor necrosis factor alpha (TNF‐α) induced cellular and mitochondrial ROS generation [69]. TNF‐α led to decreased MITF‐M expression (a transcriptional regulator of melanogenesis), and increased ICAM1, TNFR1, and IL‐6 levels [69]. An increase in TNF‐α leads to an increase in CD4+ and CD8+ cells [28].

#### 3.5.4 Nuclear Factor Eryhtroid‐2

Nuclear factor erythroid‐2 (Nrf2) signaling activity, which is essential to combat oxidative stress, was disordered in vitiligo. Nrf2 genetic variation, particularly rs35652124, was associated with vitiligo [44]. In oxidative stress, Nrf2 accumulates and translocates to the nucleus, leading to the expression of antioxidant response gene [20].

#### 3.5.5 B‐Cell Activating Factor

B‐cell activating factor (BAFF) is a cytokine essential for B‐cell survival and regulation of B‐cell surface protein expression. Overexpression of BAFF leads to the emergence of autoreactive B cells, breakdown of self‐tolerance, and the production of autoantibodies against melanocytes. These autoantibodies may activate CD4+ T cells, further exacerbating the immune response against melanocytes [99].

### 3.6 Pathways

#### 3.6.1 Fas/FasL

Fas/FasL pathway was involved in CD8+ T cells and NK cells mediated melanocyte apoptosis [56, 65, 71]. Fas receptor belongs to the TNF receptor superfamily, and when interacting with its ligand FasL, initiates a cascade leading to apoptosis [56].

#### 3.6.2 Perforin/Granzyme

Melanocyte apoptosis mediated by CD8+ T cells and NK cells was mediated by the perforin‐granzyme pathway [65]. Perforin induced cell death via hypomethylation of specific CpG sites on CD8+ T cells [74].

#### 3.6.3 IL‐1β/IL‐1R

Oxidative stress led to calcium influx and mitochondrial ROS accumulation. This activates the NLRP3 inflammasome in keratinocytes [86]. IL‐1β/IL‐1R signaling via the NLRP3 inflammasome enhanced CD4+ and CD8+ T‐cell functions. This includes increased production of IL‐17A/F and IFN‐γ in CD4+ T cells and overexpression of CXCR3, CXCR6, and IFN‐γ on CD8+ T cells. TRPM2, known for mitochondria‐dependent apoptosis of melanocytes, was implicated in this pathway. IL‐1β promotes the expression of CXCL10 and CXCL16 in keratinocytes, contributing to CD8+ T‐cell infiltration [86]. Melanocytes are shown to express the chemokine receptor CXCR3, particularly the CXCR3B isoform [14]. CXCL10 can induce melanocyte apoptosis [14].

#### 3.6.4 IRE1/XBP1 Pathway

IRE1/XBP1 pathway influenced the differentiation of Th17 cells, which produce IL‐17A [84].

#### 3.6.5 Endoplasmic Reticulum Stress

Oxidative stress led to an increase in endoplasmic reticulum (ER) stress‐related proteins which were PERK, eIF2α, unfolded protein response (UPR) and CHOP, and a decrease in RIPK1 [53, 87]. ER stress induced melanocyte apoptosis [84, 87].

#### 3.6.6 Cytochrome C‐Mediated Caspase Pathway

Oxidative stress stimulated the activation of JNK and MAPK, initiating the cytochrome c‐mediated caspase pathway, resulting in premature melanocyte senescence and apoptosis [8].

#### 3.6.7 ERK/MAPK Pathway

Downregulation of long noncoding RNA TUG1 inhibited melanogenesis by affecting the phosphorylation of the ERK/MAPK signaling pathway, leading to a reduction in MITF [90].

#### 3.6.8 Tyrosinase‐Dependent Pathway

Tyrosinase‐dependent pathway induced death of melanophores [11]. Tyrosinase, a protein crucial for melanin synthesis, is one of the antigens responsible for the production of antibodies in vitiligo [107].

## 4 Treatment

Current treatment for vitiligo can be divided into six approaches which are oral, topical, phototherapy, invasive, surgical, and combination therapies. The treatments presented here have undergone clinical trials and were conducted on different vitiligo conditions with three variables: vitiligo characteristics, age, and ethnic groups. Different groups of people respond differently to vitiligo treatment. This scoping review only reports successful clinical trials. Effective treatment is characterized as complete resolution of symptoms [110]. Vitiligo Area Scoring Index (VASI) score, a quantitative parametric score, was introduced by Hamzavi et al. [111] measure the outcome of an anti‐vitiligo treatment. The formula for VASI score is as follows:

$$\begin{array}{cc} \text{VASI Score}\; & =\sum \mathrm{All}\;\text{body sites}\;\left(\text{Hand units}\right)\\ & \times \left(\text{Residual depigmentation}\right) \end{array}$$

Extent of repigmentation was classified into 5: complete, excellent, good, moderate, and poor. Complete repigmentation means repigmentation of 100% of the lesions, excellent repigmentation means repigmentation of 76%–100% of the lesions, good repigmentation means repigmentation of 51%–75% of the lesions, moderate repigmentation means repigmentation of 26%–50% of the lesions, while poor repigmentation means repigmentation of 1%–25% of the lesions [112]. The formula for efficacy rate is as follows [113]:

$$\begin{array}{cc} \text{Efficacy rate}\; & =\%\text{of excellent repigmentation}\\ & +\%\text{of good repigmentation} \end{array}$$

Table 2 shows the proposed available treatments for vitiligo.

**TABLE 2:** Proposed available treatments for vitiligo.

Therapy	Vitiligo characteristics	Patients number	Age range (years)	Clinical results	Evaluation time	References
*Oral*						
Hydroxychloroquine	Nonsegmental vitiligo	15	More than 16	Repigmentation on all the body regions was significantly higher than the baseline	3 months	[114]
Ritlecitinib	Active nonsegmental vitiligo	364	18–65	Significant facial VASI (F‐VASI) score with 50 mg ritlecitinib. Accelerated improvement was observed after treatment with ritlecitinib 200/50 mg in the extension period	48 weeks	[115]
Preparation containing coffee and sunflower seed	Plurisgemnetal vitiligo	1	28	Repigmentation began to appear in the third month. Partial repigmentation on the right shoulder, arm, sinus, neck, and chest. The non‐pigmented patches remained stabilized without reports of new patches until the last patient observation	3 months	[116]
Tofacitinib	Bilateral upper eyelids with associated leukotrichia	1	17	Partial repigmentation of the bilateral upper eyelids. The bilateral eyelashes showed near‐complete repigmentation	2 months	[117]
Individualized homeopathic medicine (IHM)		60	Not specified	Higher mean reductions compared to placebo	6 months	[118]
Vitamin D		101	Not specified	Patients with sufficient 25(OH)D levels achieved a significantly higher degree of repigmentation	6 months	[119]
Apremilast	Nonsegmental vitiligo	13	19–60	Significant mean reduction of VASI	3 months	[120]
Baricitinib	Nonsegmental vitiligo	4	21–34	Significant repigmentation without obvious side effects. Depigmentation occurred in 2 patients at the 3‐month follow‐up	12 weeks	[121]
*Topical*						
Ruxolitinib	Nonsegmental vitiligo [122, 123, 124]	33 [125], 674 [122, 123, 124]	23–56 [122], More than 12 [124], 24–55 [123]	1 T‐VASI50 responders in the head/neck region were 60.0%, 52.9% and 52.6% in upper and lower extremities, respectively, 15.0% in hands and 29.4% in feet [125]. 50% decrease of F‐VASI at week‐24 and 30% had a decrease of at least 75%, with improvement through week 52. At Week 24, F‐VASI75 was achieved by 30.1% of patients [124]. 50.3% of patients who applied ruxolitinib cream from day 1 achieved F‐VASI75 [123]	52 weeks [122, 123, 125], 24 weeks [124]	[125], [122], [124], [123]
Depigmentation with monobenzyl ether of hydroquinone (MBEH)	Nonsegmental vitiligo	39	More than 12	MBEH 20% is suitable for face whereas MBEH 40% is suitable for hand and areas of thick skin	6 months	[126]
Turmeric cream		24	Not specified	Significant repigmentation	4 months	[127]
Ayurvedic treatment		1 [128, 129]	11 [129], 55 [128]	Near‐complete repigmentation [129]. Perifollicular pigmentation in 5 patches and complete repigmentation in 12 patches [128]	10 months [129], 6 months [128]	[129], [128]
Depigmentation with 88% phenol, then 3% glutathione cream	Not specified	1	74	Effective depigmentation lasting 2 weeks after each application	3 weeks	[130]
Topical preparation containing Activity Melanoma Inhibitory (MIA)	Nonsegmental vitiligo	1	50	Good repigmentation without any side effects locally or systemically	9 months	[131]
*Piper Nigrum* extract	Segmental and nonsegmental vitiligo	3	40–70	Skin repigmentation achieved	12 weeks	[132]
TCA	Stable vitiligo [133], Universal vitiligo [134]	100 [133], 50 [134]	22–65 [134]	Excellent depigmentation in 80% of cases with eyelid vitiligo, followed by the face, trunk, and extremities. Lower response rates in the hands and feet vitiligo [133]. Excellent depigmentation in 80% of cases good depigmentation in 12%, and moderate and poor depigmentation in 8% [134]	6 months [133], 10 weeks [134]	[133], [134]
Topical MTX	Not specified	1	23	Initial regimentation was noted at week 4. Further regimentation progressed with significant improvement at week 10. No local or systemic side effects were noted	12 weeks	[135]
Topical crisaborole	Stable vitiligo	1	Not specified	Skin repigmentation achieved		[136]
Topical *Nigella sativa*	Not specified	33	20–58	Excellent and good repigmentation in 43.5% with facial vitiligo, 43.8% with hand vitiligo, and 87.5% with genital vitiligo	6 months	[137]
*Phototherapy*						
Cold atmospheric plasma	Active focal vitiligo	20	More than 12	Partial and complete repigmentation in 80% and 20% of vitiligo lesions, respectively without hyperpigmentation or other adverse events	8 months	[138]
NB‐UVB phototherapy	Segmental and nonsegmental vitiligo	31 [139], 58 [140]	7–67 [139], 14–77 [140]	38.7% of patients achieved > 50% VASI changes while 41.9% achieved 25%–50% VASI changes. Total good and very good response to therapy significantly increased with prolonged treatment, increasing from 19.4% to 64.5% and 80.6% after 2, 4 and 6 months, respectively. Localized NSV obtained good and very good response significantly more than generalized NSV (55.6% vs. 18.2%) [139]. NSV and SV demonstrated overall improvement in Vitiligo Area Scoring Index (VASI) score of −50.0% ± 31.0% and −40.0% ± 28.3%, respectively. Persistence of repigmentation was observed in approximately 80% of cases at 1 year after discontinuation of NB‐UVB [140]	6 months [139], 12 months [140]	[139], [140]
308‐nm LED	Stable vitiligo	70		The efficacy rate was 49.49%		[141]
Low‐intensity pulsed ultrasound (LIPUS)	Stable NSV	27	20–75	Repigmentation in 38.5% with truncal vitiligo, but none with facial vitiligo	24 weeks	[142]
Yttrium Aluminum Garnet (YAG) laser	Stable vitiligo	18	Not specified	50% repigmentation in 88.8% of facial; 77.7% of dorsal hand; 75% of limb; and 25% of finger vitiligo	3 months	[143]
Home‐based NB‐UVB	NSV	10	21–54	Excellent and good repigmentation in 10% and 60%, respectively	6 months	[112]
*Invasive*						
Filiform fire needle therapy	SV and NSV	77	6–79	Excellent and good repigmentation in 44.15% and 19.48%, respectively	12 weeks	[144]
Micropigmentation	Stable vitiligo	14	10–66	80.0% showed excellent overall color matching	3 months	[145]
Biorevitalizant NCTF135	NSV	7	25–54	Partial or complete repigmentation in 100% of cases	5 weeks	[146]
PRP	Stable vitiligo	10	Mean: 36.2	Excellent and good repigmentation in 20% and 20% of cases, respectively. No recurrence of depigmentation after a mean follow‐up of 6 months	24 months	[147]
*Surgical*						
Suction blister epidermal graft (SBEG)	Penile vitiligo	1	32	Complete repigmentation	3 months	[148]
ReCell	Nipple‐areola vitiligo	18	15–42	Mean repigmentation of 96.1% ± 3.5%	12 months	[149]
Autologous noncultured and trypsinized melanocyte growth	SV and NSV	28	15–60	Good‐to‐excellent repigmentation was seen in the face and neck, trunk, upper extremity, and genitals in 57.4%, 20.4%, 16.7%, and 5.5% patients, respectively	18 months	[150]
Autologous noncultured melanocyte–keratinocyte cell suspension	Stable NSV	39	20–48	85% good repigmentation	12 months	[151]
Autologous melanocyte–keratinocyte grafting	Refractory vitiligo	32	Mean: 28.03 ± 5.83	Good and excellent repigmentation in 18.2% and 26.3% of cases, respectively	18 months	[152]
Automated epidermal micrograft harvesting	Stable SV and NSV	34		Significant improvement of VASI score. Comparable outcomes between SV and NSV	12 months	[153]
Melanocyte–keratinocyte transplantation	SV and NSV	25	Mean: 32.4	Significant improvement of VASI score	12 months	[154]
Autologous noncultured epidermal cell suspension	Stable vitiligo	41	8–50	80.5% showed good‐to‐excellent response; with 17.1% showed complete or almost complete repigmentation	9 months	[155]
Autologous noncultured epidermal cellular grafting	Stable SV	1	Not specified	Skin repigmentation achieved	12 weeks	[156]
Noncultured epidermal cellular grafting (NCEG)	Stable vitiligo	38	17–67	25.0% repigmentation	12 months	[157]
NCES	Stable vitiligo	134		Excellent and good repigmentation in 82.9% and 10.0% of cases, respectively	6 months	[158]
PRP‐enriched epidermal suspension transplant	Stable vitiligo	10	Majority 20–30	60% showed excellent response; of which 50% showed complete repigmentation in 8 weeks	6 months	[159]
*Combination*						
NB‐UVB phototherapy combined with 1.5% ruxolitinib cream	Not specified	19	Mean = 47.2	Skin repigmentation achieved	104 weeks	[160]
NB‐UVB phototherapy combined with systemic acitretin		20	Not specified	Early onset of repigmentation. E‐cadherin levels were improved		[161]
Epidermal keratinocyte–melanocyte cells suspension combined with microneedling	Facial vitiligo	15	18–45	Significant repigmentation	6 months	[162]
Monocyte‐rich platelet‐rich plasma (PRP) combined with 1927 nm fraxel laser and a 308 nm excimer laser	Stable NSV	27	20–69	Repigmentation in 59% of cases with reduced VES	10 months	[163]
Noncultured melanocyte–keratinocyte transplantation combined with motorized micropunch grafting	Not specified	15	12–67	Significant repigmentation	4 months	[164]
NB‐UVB phototherapy combined with subcutaneous afamelanotide	NSV	18	Not specified	Significant reduction in VASI score in head and neck, hands, upper extremities, trunk, and lower extremities vitiligo	13 months	[165]
308‐nm excimer laser combined with 0.1% tacrolimus	SV	50	3–60	Complete and excellent repigmentation in 35.6% and 42.2% of cases		[166]
308‐nm excimer laser combined with tacrolimus and calcipotriene	Stable lip vitiligo	3	22, 52, 46	Almost near‐total repigmentation	26, weeks	[167]
NB‐UVB phototherapy combined with tofacitinib	SV [168], NSV [169]	1 [168], 50 [169]	4 [168]	Complete repigmentation [168], Significantly higher repigmentation [169]	6 months [168], 8 weeks [169]	[168], [169]
NB‐UVB combined with simvastatin		1	34	Skin repigmentation achieved		[170]
NB‐UVB phototherapy combined with oral baricitinib	NSV	2	17 and 56	Excellent repigmentation with good tolerance	6 months	[171]
NB‐UVB phototherapy combined with autologous micrografts	Stable vitiligo	20	25–51	Repigmentation rate of 64.6%	6 months	[172]
Camouflage combined with psychotherapy	Active and stable vitiligo	238	18–60	Serum levels of neuropeptide‐Y and melanin‐concentrating hormone significantly decreased, and serum level of adrenocorticotropic hormone increased	4 weeks	[173]
308‐nm excimer laser combined with 0.03% tacrolimus ointment	NSV	73	Not specified	Complete repigmentation of 36.2%. The efficiency rate was 81.9%	24 months	[174]
PRP combined with excimer laser	Stable NSV	52	18–40	In PRP/excimer laser group, 60% 13%, and 27% of patients had perifollicular repigmentation, marginal repigmentation, and both repigmentation patterns, respectively	11 months	[175]
CO_2_ laser combined with cultured epidermal autografts	Stable vitiligo	11	13–58	The mean percentage of repigmentation was 63.3%	12 months	[176]
Fractional ablative CO_2_ combined with 5‐FU	NSV	30	Not specified	Significant improvement of VESTA score	12 weeks	[177]
Topical tofacitinib combined with phototherapy	Stable NSV	1	17	Significant repigmentation of the forehead, nose, eyes, and lips	9 months	[178]
Noncultured, nontrypsinized epidermal cell grafting homogenized with plasma gel combined with NB‐UVB phototherapy	Stable vitiligo	40	19–44	Complete and excellent repigmentation in 35% and 30% of cases, respectively	4 months	[179]
FCO_2_ laser combined with 1% phenytoin cream	Stable NSV	25	18–59	VASI score before and after treatment was 0.50 and 1.48 in acral areas, 0.45 and 2.04 in upper extremities and 0.79 and 3.39 in trunk, respectively	6 months	[180]
Microneedling combined with 5‐FU and oral corticosteroid	Not specified	1	10	Complete repigmentation of the knees. No recurrence was noted at the week 52	16 weeks	[181]
NB‐UVB phototherapy combined with surrounding needling	Stable NSV	17	20–80	Significant improvement of VASI score	12 weeks	[182]
NB‐UVB phototherapy combined with vitamin A and E	NSV	46	31–52	Skin repigmentation was achieved	4 months	[183]
NB‐UVB phototherapy combined with triamcinolone	NSV	20	Not specified	Significant repigmentation	12 months	[184]
Topical band‐pass filter cream assisted home‐based NB‐UVB	Stable SV	1	32	Good repigmentation without adverse effects with persistent efficacy over the next 12 weeks	12 weeks	[185]
NB‐UVB phototherapy combined with autologous mini‐graft transplantation (mMG)	Stable NSV	14	6–77	Excellent repigmentation was observed	24 months	[186]
Triple therapy of Er:YAG laser ablation, 5‐FU, and NB‐UVB phototherapy	Stable symmetrical vitiligo	40	12–60	Excellent and good repigmentation was observed in 30% and 37.5% of cases, respectively	4 months	[187]
FCO_2_ laser combined with NB‐UVB phototherapy	Stable symmetrical vitiligo	32	18–35	Excellent and good repigmentation was observed in 25% and 6.3% of cases, respectively	4 months	[188]
308‐nm excimer laser combined with halometasone versus monotherapy	Not specified	233	Pediatric	70.39% had complete repigmentation	12 weeks	[189]
Topical corticosteroid combined with topical calcineurin inhibitors	Not specified	110	4–10	The overall > 50% repigmentation rate was 64.5%	86 months	[190]
Tofacitinib combined with 308‐nm excimer laser	NSV		Not specified			[191]
Skin autografts combined with PUVA		28	19–35	Complete and excellent repigmentation in 19 and 15 foci, respectively	12 months	[192]
*Comparison*						
Comparison of autologous noncultured melanocyte transfer vs. split‐thickness skin graft (STSG)	Stable vitiligo	50	11–40	62% patients showed good repigmentation. Patches over face, lips, trunk and legs showed good repigmentation; however, patches over acral areas and bony prominences had poor repigmentation	6 months	[193]
Comparison of microneedling combined with 5‐FU versus monotherapy	Stable SV	46	10–50	Excellent repigmentation of 48.6% in microneedling/5‐FU compared to 16.9% in microneedling monotherapy	6 months	[194]
Comparison of STSG versus autologous noncultured nontrypsinized epidermal cell transplant, also known as Jodhpur technique (JT)	Stable vitiligo	32	10–50	Excellent and good repigmentation in 72.5% and 95% of cases in the JT group compared to 40% and 83.75% in the STSG group, respectively	20 weeks	[195]
Comparison of follicular unit transplantation (FUT) versus JT	Stable focal segmental or NSV	30	More than 10	Excellent and good repigmentation in 70% and 18% of cases in FUT group compared to 72% and 26% in JT group	20 weeks	[196]
Comparison of 5 mg betamethasone oral mini‐pulse (OMP) versus azathioprine	Active NSV	55	More than 18	2, 2, and 9 patients in the OMP group had more than 20%, 10% to 20% and 5% to 10% repigmentation, respectively, whereas only 2 patients in the azathioprine group had 10% to 20% repigmentation, with the remaining patients having no or less than 5% repigmentation	6 months	[197]
Comparison of NB‐UVB phototherapy combined with 1% pimecrolimus versus monotherapy	Segmental and vitiligo vulgaris	114	11–17	Significant improvement with combination therapy. Repigmentation rate for CNI monotherapy was 67.4%, 80.5% for phototherapy, and 93.7% for combined therapy		[198]
Comparison of NB‐UVB phototherapy combined with heterologous type I collagen (HTIC) versus monotherapy	NSV	5	More than 18	Mean repigmentation rate was 70.5% in the NB‐UVB/HTIC group compared to 16.5% in NB‐UVB monotherapy	12 weeks	[199]
Comparison of microneedling combined with 5‐FU versus monotherapy	Stable SV [200, 201], SV and NSV [202], NSV [203]	40 [200], 60 [201], 22 [202], 50 [203]	More than 10 [201], 8–70 [202]	Significant repigmentation in combination therapy compared to monotherapy [200]. Initiation of repigmentation started at 1 month in 65% in microneedling/5‐FU groups compared to 38.7% in 5‐FU monotherapy. Excellent repigmentation was observed in 47% in microneedling/5‐FU groups compared to 4.3% in 5‐FU monotherapy [201]. 40% and 26.6% achieved excellent and good repigmentation [202]. No repigmentation in microneedling monotherapy compared to 76% in microneedling/5‐FU [203]	4 months [200], 6 months [201, 202], 3 months [203]	[200], [201], [202], [203]
Comparison of OMP versus oral cyclosporine	Active NSV	50	16–60	Arrest of disease progression (ADP) was attained in 84% in OMP group and 88% patients in cyclosporine group. However, mean time to achieve ADP was significantly lower in cyclosporine group as compared to ADP group (10.92 [4.12] weeks vs. 13.90 [3.92] weeks)	6 months	[204]
Comparison of 0.1% tacrolimus ointment versus 0.05% clobetasol propionate	Not specified	162	15–40	In tacrolimus group, 51.9% had complete repigmentation while inn clobetasol group, 58% achieved skin repigmentation	12 weeks	[110]
Comparison of NB‐UVB phototherapy alone versus combination with oral *Silybum marianum*	Not specified	34	15–67	Improvement of VASI score in both groups	9 months	[205]
Comparison of microneedling combined with either TCA, 5‐FU, or pimecrolimus	Stable NSV	75	Not specified	Microneedling/TCA had more significant repigmentation compared to other groups	12 weeks	[206]
Comparison of dexamethasone OMP versus mycophenolate mofetil	Active NSV	50	19–59	No significant difference was observed in VASI scores	9 months	[207]
Comparison of combination of OMP dexamethasone and methotrexate (MTX) versus monotherapy	Active vitiligo	42	More than 18	71.4% perilesional pigmentation in combination treatment compared with 42.9% in dexamethasone group. Frequency of intralesional repigmentation showed a statistically significant increase in oral MTX alone (50%) and combination therapy (42.9%) groups compared with that in OMP dexamethasone alone (14.3%) group	3 months	[208]
Comparison of triple therapy of NB‐UVB phototherapy, microneedling and topical latanoprost versus double therapy	Stable NSV	50	16–49	Mean repigmentation rate of 44.15% ± 33.91% in NB‐UVB/microneedling/latanoprost compared to 19.25% ± 19.89% in NB‐UVB/microneedling	12 months	[209]
Comparison of microneedling alone versus mini‐punch grafting (MPG) alone versus combination therapy	Stable NSV	20	Not specified	Combination therapy observed median repigmentation of 49.6%, followed by 38.5% in MPG and 33.4% in microneedling	6 months	[210]
Comparison of triple therapy of microneedling, topical 5‐FU, and excimer light versus excimer light monotherapy	Stable NSV	33	3–56	Triple therapy showed good‐to‐excellent repigmentation in 18.2% of cases, while excimer light monotherapy showed good repigmentation in 3% of cases	6 months	[211]
Comparison of electrocautery (EC) needling combined with 308‐nm excimer light versus monotherapy	Stable NSV	30	Not specified	Mean repigmentation of 34.86% in EC needling, 31.29% in excimer light and 50.95% in combination therapy	12 weeks	[212]
Comparison of combination of UVB microphototherapy (Bioskin) with VITILSI gel versus monotherapy	Not specified	10	18–49	Repigmentation rate of 28% in Bioskin group, 19% in VITILSI group, 41% in Bioskin/VITILSI group and null in placebo	8 weeks	[213]
Comparison of 308‐nm excimer light versus 311‐nm NB‐UVB phototherapy	Stable vitiligo	36	18–65	Significant repigmentation in light group. 25% treated with 308‐nm excimer light and 13.89% treated with 311‐nm NB‐UVB achieved excellent repigmentation	12 months	[214]
Comparison of microneedling combined with either 5% 5‐FU or 0.1% tacrolimus	Stable vitiligo	30	12–60	Significantly higher repigmentation with 5‐FU compared to tacrolimus. Good‐to‐excellent repigmentation in 76.7% of the cases with 5‐FU compared to 63.7% with tacrolimus	6 months	[215]
Comparison of FCO_2_ laser with either NB‐UVB phototherapy, topical tacrolimus, or topical calcipotriol	Stable NSV	30	15–57	In laser/tacrolimus group, excellent and good repigmentation were observed in 30% and 30% of cases, respectively. In laser/calcipotriol excellent and good repigmentation were observed in 10% and 30% of cases, respectively. In laser/NB‐UVB group, excellent and good repigmentation were observed in 40% and 40% of cases, respectively	6 months	[216]
Comparison of NB‐UVB phototherapy combined with carboxytherapy versus monotherapy with NB‐UVB	Stable NSV	28	25–40	Excellent repigmentation was observed in 37% of the patients in combination group compared to 0% in the monotherapy group	8 months	[217]
Comparison of combination of NCES with microneedling and 5‐FU versus monotherapy	Stable acral vitiligo	50	Not specified	Excellent repigmentation was observed in 84% of the patients in combination group compared to 40% in the monotherapy group	24 weeks	[218]
Comparison of FUT versus MPG	Stable localized and SV	25	Not specified	MPG group showed a significant higher and earlier repigmentation compared to FUT group	9 months	[219]
Comparison of combination of 308‐nm excimer laser and topical tacrolimus versus 308‐nm excimer laser monotherapy	Periocular vitiligo	58	Not specified	In the laser group, excellent and good repigmentation was observed in 11% and 36% of cases. In the laser/tacrolimus group, excellent and good repigmentation was observed in 50% and 23% of cases		[113]
Comparison of PRP‐suspended NCES versus Ringer's lactate (RL)‐suspended NCES	Stable vitiligo	40	Not specified	Significantly higher repigmentation in PRP group compared to RL group	6 months	[220]
Comparison of 5‐FU injection combined with FRCO_2_ versus 5‐FU monotherapy	Stable vitiligo	40	Not specified	Repigmentation was demonstrated in 90% of patients in FRCO_2_/5‐FU compared to 85% in 5‐FU group. Good‐to‐excellent repigmentation were observed in 50% of patients in FRCO_2_/5‐FU groups and 55% in 5‐FU groups		[221]
Comparison of NB‐UVB phototherapy combined with MPG versus SBEG	Stable vitiligo	23	17–32	Repigmentation rate was 98.7% in MPG, 98% in SBEG (blister for recipient site) and 99.3% in SBEG (dermabrasion for recipient site)	3 months	[222]
Comparison of follicular unit extraction (FUE) combined with either topical calcipotriol betamethasone dipropionate (CBD) or NB‐UVB versus monotherapy	Stable NSV	53	10–55	The fastest onset of repigmentation was observed in both FUE/CBD and FUE/NB‐UVB in the second week (16.7%, 10.5%), respectively. However, no significant difference was detected at the end of 4 months	4 months	[223]
Comparison of triple therapy of NB‐UVB phototherapy, FCO_2_, and 0.01% topical bimatoprost versus dual therapy of NB‐UVB phototherapy and FCO_2_.	Stable NSV	15	Not specified	Significantly higher repigmentation in triple therapy group compared to dual therapy	12 weeks	[224]
Comparison of manual dermabrasion (MD) versus electrofulguration‐assisted dermabrasion in NCES	Stable vitiligo	26	10–45	Excellent repigmentation was observed in 69.3% in MD group compared to 73.1% in EF group. EF achieved successful repigmentation earlier as compared to MD (9.4 weeks vs. 11.4 weeks)	24 weeks	[225]
Comparison of SEBG versus automated blister epidermal micrograft (ABEM)	Stable SV and NSV	75	7–70	Excellent and good repigmentation was observed in 76% and 8% of the SBEG group compared to 39% and 21% in ABEM group	3 months	[226]
Comparison of NB‐UVB phototherapy combined with topical corticosteroid (TCS) versus monotherapy	NSV	517	More than 5	Repigmentation rate of 15% was observed in combination treatment, followed with 8% in NB‐UVB and 3% in TCS	9 months	[227]
Comparison of pimecrolimus combined with microneedling versus monotherapy	Stable vitiligo	15	18–60	Excellent repigmentation was observed in 6.7% in combination therapy. No significant repigmentation was observed in the pimecrolimus group	6 months	[228]
Comparison of combination of 0.1% tacrolimus ointment with basic fibroblast growth factor (bFGF) versus monotherapy	Stable vitiligo	84	25–52	Good‐to‐excellent repigmentation was significantly higher in BFGF/tacrolimus 0.1% group (22.5%) compared to tacrolimus monotherapy (6.8%)	6 months	[229]
Comparison of 5‐FU versus triamcinolone acetonide	Stable NSV	60	18–59	Median repigmentation of 52.27 was observed in 5‐FU group compared to 13.86 with triamcinolone and 17.15 with the drug mixture group During follow‐up, the vitiliginous patches continued to repigment for 6 months in 5‐FU and the drug mixture groups. The repigmentation stopped 1 month after the last session in the triamcinolone group	6 months	[230]
Comparison of combination of 308‐nm monochromatic excimer light with 0.1% topical tacrolimus versus monotherapy	Symmetrical vitiligo	30	12–57	Lesions located on face and trunk in 9.31% combination therapy achieved complete repigmentation versus 47.8% in monotherapy. 47.1% lesions located on extremities and acral area in group combination therapy achieved good repigmentation compared to 11.8% in monotherapy	30 weeks	[231]
Comparison of suction blistering technique versus mini‐punch technique versus hair follicle technique	Stable vitiligo	30	16–60	The mean repigmentation rate in the suction blister, mini‐punch and hair follicle techniques were 90% ± 10.54%, 57% ± 18.88%, and 18% ± 7.89%, respectively	6 months	[232]
Comparison of combination of microneedling with calcipotriol and betamethasone versus combination with tacrolimus	Stable vitiligo	25	11–36	Excellent repigmentation observed in 60% of the patients in microneedling/calcipotrio/betamethasone group compared to 32% in microneedling/tacrolimus group. It was effective in the most resistant sites of vitiligo such as: elbows, knees, extremities, and acral area	9 months	[233]
Comparison of NB‐UVB phototherapy combined with Vitilinex versus monotherapy	Stable or active SV	62	18–58	In Vitilinex monotherapy, 39% achieved excellent repigmentation with 22% patients experiencing complete repigmentation. In Vitilinex/NB‐UVB, 69.5% achieved excellent repigmentation with 75% experiencing complete repigmentation. In NB‐UVB monotherapy, 37.5% achieved excellent repigmentation with 33.33% patients experiencing complete repigmentation	12 weeks	[234]
Comparison of dermaroller, dermabrasion, and cryoblister noncultured epidermal cell suspension	Stable vitiligo	36	18–60	Dermabrasion and cryoblister techniques showed excellent repigmentation in 55.6% and 47.2% of cases, respectively. However, dermabrasion was superior to cryoblister in terms of rapidity (65% vs. 32.5% at 4 weeks) and color match (47.2% vs. 19.4%). Dermaroller had poor repigmentation outcomes compared to both dermabrasion and cryoblister	12 weeks	[235]
Comparison of combination of oral PUVA therapy with topical bFGF related decapeptide versus monotherapy	Stable NSV	120	18–65	Good‐to‐excellent repigmentation was achieved in 61.8% from combination group compared to 30.2% from oral PUVA group. Complete repigmentation was observed in 5.5% compared to 0% in monotherapy group	6 months	[236]
Comparison of FCO_2_ laser combined with PRP versus monotherapy	Stable NSV	66	18–50	Significant reduction in VASI score in the combination group compared to monotherapy group	3 months	[237]
Comparison of targeted UVB (TUVB) versus excimer light	SV	40	2–45	Excellent and good repigmentation in 68.1% and 82.6% lesions in excimer light compared to 46.4% and 76.3% in TUVB group	15 weeks	[238]
Comparison of combination of MTX with either NB‐UVB phototherapy or excimer laser versus monotherapy	NSV	48	6–62	Mean repigmentation of 49.7% ± 33.5% in MTX/NB‐UVB group compared to (9.3% ± 20.7%) in MTX monotherapy, and 39.9% ± 33.8% in MTX/excimer light group	4 months	[239]
Comparison of PRP combined with 308‐nm excimer laser versus monotherapy	Localized stable vitiligo	60	18–65	VASI score of laser/PRP group was significantly higher than that of monotherapy. Excellent and good repigmentation was observed in 40% and 40% of cases, respectively	3 months	[240]
Comparison of 0.03% tacrolimus versus hydrocortisone	NSV	63	3–75	Repigmentation was observed in 45.2% of patients in tacrolimus group versus 0.0% in hydrocortisone group. Excellent and good repigmentation in 3.2% and 9.7% of patients, respectively	24 weeks	[241]
Comparison of combination of UVB phototherapy with CO_2_‐UVB versus monotherapy	Stable vitiligo	10	Not specified	Significant repigmentation. After 5 years, one patient lost his partial response and two patients developed light hyperpigmentation on both sides	5 years	[242]
Comparison of combination of NB‐UVB phototherapy combined with topical oleyl alcohol‐based transethosomal 8‐methoxypsoralen versus monotherapy	Acral vitiligo	15	18–63	Significant improvement in VESTA score compared to monotherapy	12 weeks	[243]
Comparison of combination of 5‐FU with Erbium:YAG (Er:YAG) versus monotherapy	Stable NSV	30	15–59	The mean repigmentation of Er:YAG/5‐FU group is 12% ± 7% compared to 1.4% ± 0.8% in 5‐FU group	9 months	[244]
Combination of dermabrasion with either dinoprostone or tacrolimus.	Stable vitiligo	40	Not specified	Tacrolimus group showed slightly better response, whereas the side effect profile was better for group dinoprostone		[245]

### 4.1 Oral

Currently, successful clinical trials for oral treatments of vitiligo include prescription medications, herbal preparations, vitamin D, and homeopathy. The prescription medications include antirheumatic medication hydroxychloroquine, immunosuppressants ritlecitinib, tofacitinib, and baricitinib, and phosphodiesterase inhibitors apremilast. A case report of successful repigmentation using a herbal preparation comprised of coffee and sunflower seed was reported in a 28‐year‐old patient. In a comparison trial of OMP versus azathioprine, 15.38% of patients receiving 5 mg betamethasone OMP had more than 20% repigmentation, compared to none of the patients receiving azathioprine [197]. When compared with mycophenolate mofetil, both OMP and mycophenolate mofetil halt actively spreading vitiligo after 45–50 days. However, relapse occurred much higher and significantly earlier with mycophenolate mofetil [207]. Comparing OMP with cyclosporine, arrest of disease progression was achieved in 88% of the cyclosporine group compared to 84% in the OMP group. However, the cyclosporine group's mean time to achieve ADP was significantly lower [204].

### 4.2 Topical

Seven distinct topical preparations—immunosuppressant, herbal preparation, activity melanoma inhibitory (MIA) based cream, corticosteroid, non‐steroidal anti‐inflammatory drug (NSAID), anti‐metabolite, and Ayurvedic medicine were—documented in this scoping review. Potent immunosuppressant ruxolitinib demonstrated successful repigmentation in four different reports [122, 123, 125, 160]. With 0.03% tacrolimus, 45.2% repigmentation was observed [241]. Herbal formulations, which were turmeric [127], *piper nigrum* [132], and *Nigella sativa* [137] based cream, improved the repigmentation significantly compared to the placebo group. Without any side effects, a topical treatment containing MIA demonstrated 50%–80% repigmentation from the baseline [131]. When clobetasol and tacrolimus were compared, the clobetasol group reported 58% effective treatment compared to 51.9% in the tacrolimus group [110]. Crisaborole, an NSAID, proved effective in treating vitiligo [136]. In addition, significant repigmentation was also recorded by the anti‐metabolite MTX on week 10 [135].

Ayurvedic medicine is a traditional medicine that originated in India. Treatment options include yoga, acupuncture, herbal medicine, massage, and dietary adjustments. Two cases managed with Ayurvedic medicine reported excellent repigmentation in 6–12 months [128, 129]. Depigmentation therapy is an alternative therapy for patients extensively affected by vitiligo, by depigmenting the whole skin instead of repigmenting it to achieve even skin tones. Recently mentioned depigmentation agents were TCA, MBEH, phenol, and glutathione. In 80% of the cases, depigmentation therapy with an analogue of acetic acid, TCA, reported more than 90% depigmentation [133, 134]. MBEH 20% was more suitable for facial vitiligo, while MBEH 40% was more suitable for areas of thick skin such as the hand [126]. Also, effective depigmentation was reported with 88% phenol combined with 3% glutathione [130].

### 4.3 Phototherapy

Eight different phototherapy procedures—cold atmospheric plasma, UVB, excimer laser, light‐emitting diodes, LIPUS, YAG laser, FCO_2_, and PUVA—were described in this scoping review. Cold atmospheric plasma reported complete repigmentation in 20% of vitiligo lesions [138]. NB‐UVB phototherapy was divided into two categories: clinic‐based and home‐based phototherapy. In terms of clinic‐based phototherapy, the rate of good repigmentation significantly increased with prolonged treatment, achieving 80.6% after 6 months [139]. Silpa‐Archa reported that the VASI score for NSV and SV after 6 months of treatments was 50.0% + 31.0% and 40.0% + 28.3%, respectively [140]. In addition, persistent repigmentation was observed in 80% of cases after one‐year discontinuation of NB‐UVB [140]. In terms of home‐based phototherapy, only one patient did not respond to the treatment, and 10% of patients had excellent repigmentation after 6 months [112].

26% of 308‐nm LED patients had excellent repigmentation while 21% reported poor pigmentation [141]. In a comparison of excimer light versus TUVB, excimer light demonstrated excellent repigmentation of 68.1% compared to 46.4% in TUVB groups [238]. In addition, 308‐nm excimer light reported 25% excellent repigmentation compared to 13.89% in 311‐nm NB‐UVB [214]. In comparison with sham sonification, 38.5% of patients with truncal vitiligo reported better repigmentation with LIPUS [142]. Facial vitiligo responded best to YAG laser followed by dorsal hand, limb, and fingers [143].

### 4.4 Invasive

This scoping review identified four invasive non‐surgical approaches: filiform fire needle therapy, biorevitalizant NCTF135 injection, intradermal 5‐fluorouracil (5‐FU), and injection of PRP. According to Huang et al. (2020), filiform fire needle therapy resulted in 44.15% excellent repigmentation and 9.09% no response [144]. With an intradermal injection of biorevitalizant, complete repigmentation was observed [146]. PRP injections showed 20% excellent repigmentation with no recurrence after 2 years [147]. Instead of an actual curative treatment, micropigmentation was carried out as an alternative to conceal the vitiliginous skin tone. According to the physician's global assessment, micropigmentation reported excellent overall color matching in 80% of cases [145]. In comparison with triamcinolone, 5‐FU reported a higher rate of repigmentation. At the 6‐month follow‐up, vitiliginous patches treated with 5‐FU continued to repigment, whereas repigmentation with triamcinolone halted after 1 month [230].

### 4.5 Surgical

17 different grafts were reported, including SBEG, ReCell, autologous noncultured trypsinized melanocyte growth (NTMG), autologous noncultured melanocyte–keratinocyte cell suspension (MKCS), autologous melanocyte–keratinocyte grafting (MKG), automated epidermal micrograft harvesting (AEMH), melanocyte–keratinocyte transplantation (MKT), NCES, NCEG, PRP‐enriched epidermal suspension transplant, single follicular transplantation (SFT), FUT, JT, MPG, bFGF, AMT, and ABEM in this scoping review. A penile vitiligo case achieved complete repigmentation without complication after 3 months treatment with SBEG [148]. According to Ezz‐Eldawla et al. (2019), the mean repigmentation rate of SBEG, MPG, and FUT was 90% ± 10.54%, 57% ± 18.88%, and 18% ± 7.89%, respectively [232]. In comparison with the ABEM group, SBEG reported excellent repigmentation of 76% compared to 39% [226]. When compared to FUT, MPG demonstrated significantly higher and earlier repigmentation, similar to results from Ezz‐Eldawla et al. (2019) [219]. In contrast to Ezz‐Eldawla et al. (2019), the repigmentation rate was high in MPG, which was 98.7%, 98% in SBEG (blister for recipient site) and 99.3% in SBEG (dermabrasion for recipient site) [222]. Nipple‐areola vitiligo cases treated with ReCell achieved a 96.1% ± 3.5% mean repigmentation rate after 12 months [149]. In terms of NTMG, the face and neck were the first areas to exhibit excellent repigmentation, followed by the trunk, upper extremity, and genitalia [150]. MKCS reported 85% good repigmentation [151], while autologous MKG showed 18.2% excellent repigmentation and 24.2% poor repigmentation [152]. Following AEMH treatment, analysis utilizing VASI scores showed improvement from 96.25 ± 8.59 to 48.30 ± 28.16 [153]. At the 12‐month evaluation, improvement of VASI scores was also demonstrated in MKT [154].

In terms of NCES, Chen et al. (2021) reported 82.9% excellent repigmentation [158] while Dev et al. (2021) reported 55.77% excellent repigmentation [225]. Furthermore, Liu et al. (2021) reported 17.1% complete repigmentation [155]. Comparing PRP‐suspended NCES with Ringer's lactate‐suspended NCES, the former demonstrated significantly higher repigmentation compared to the latter [220]. The dermabrasion technique for NCES reported a better color match and more rapid and excellent repigmentation of 55.6% compared to 47.2% using cryoblister technique, while the dermaroller technique had the worst repigmentation [235]. When electrofulguration‐assisted dermabrasion and manual dermabrasion for NCES were compared, the former reported earlier and excellent repigmentation in 73.1% compared to 69.3% in the latter [225] 60% of patients who received a transplant of PRP‐enriched epidermal suspension showed excellent repigmentation [159].

In terms of NCEG, Doolan et al. (2022) observed cost‐effective sufficient skin repigmentation [156]. AMT reported 62% good repigmentation and 18% poor repigmentation. Patches covering the face, lips, trunk, and legs showed good repigmentation, while patches covering acral areas and bony prominences displayed poor repigmentation [193]. The Jodhpur technique (JT), also known as noncultured nontrypsinized epidermal cell transplant, exhibited excellent repigmentation of 72.5% compared to 40% in STSG [195]. Compared to FUT, JT observed excellent repigmentation of 72% compared to 70% in FUT [196]. In comparison with PUVA monotherapy, bFGF‐related decapeptide/PUVA reported 5.5% complete repigmentation while none in PUVA [236].

### 4.6 Double Combination Therapy

#### 4.6.1 Combination With UVB

Three different approaches were combined with UVB for vitiligo treatment—prescription medication, topical, and physical therapy. After 6 months, tofacitinib/NB‐UVB achieved complete repigmentation [168]. In addition, after 8 weeks, tofacitinib/NB‐UVB reported significantly higher repigmentation compared to control [169, 178]. Baricitinib/NB‐UVB achieved significant repigmentation in 2 case reports [171]. Only four patients experienced grade 1 or 2 adverse events when 1.5% ruxolitinib was combined with NB‐UVB [160]. Acitretin/NB‐UVB was effective in treating vitiligo with an early onset of repigmentation [161]. Simvastatin/NB‐UVB combination achieved marked repigmentation [170]. The VASI score was significantly reduced when afamelanotide and NB‐UVB were combined [165]. In comparison with monotherapy, oley alcohol‐based transethosomal 8‐methoxypsoralen combined with NB‐UVB phototherapy showed significant improvement in VESTA score [243]. MTX/NB‐UVB reported a mean repigmentation rate of 49.7 ± 33.5 compared to 39.9 ± 33.8 in MTX/excimer light and 19.3 ± 20.7 in MTX monotherapy [239].

Triamcinolone/NB‐UVB phototherapy observed significant repigmentation [184]. Topical band‐pass filter cream combined with home‐based NB‐UVB reported good repigmentation without any remarkable side effect with persistent efficacy over the next 12 weeks [185]. Comparing double therapy of NB‐UVB/1% pimecrolimus versus monotherapy, clinical efficacy for combined therapy of NB‐UVB/1% pimecrolimus was 93.7% compared to 67.4% in pimecrolimus monotherapy and 80.5% in NB‐UVB monotherapy [198]. UVB microphotherapy (Bioskin) combined with VITILSI gel reported repigmentation of 41% compared to 28% and 19% in Bioskin and VITILSI gel monotherapy [213]. In a comparison of topical corticosteroid/NB‐UVB versus monotherapy, topical corticosteroid/NB‐UVB reported excellent repigmentation of 15% compared to 8% and 3% in NB‐UVB and topical corticosteroid monotherapy [227]. Vitilinex/NB‐UVB reported an efficacy rate of 69.5%, while Vitilinex monotherapy reported an efficacy rate of 39% and 37.5% in NB‐UVB [234]. When compared with the combination of *Silybum marianum* /NB‐UVB versus monotherapy, the VASI score of *Silybum marianum* /NB‐UVB was comparable to NB‐UVB monotherapy [205]. CO_2_‐UVB combined with UVB reported significant repigmentation. However, after 5 years, 2 patients developed light hyperpigmentation [242]. Carboxytherapy/NB‐UVB reported excellent repigmentation of 37% compared to 0% in carboxytherapy [217]. Surrounding needling combined with NB‐UVB reported a significant reduction in VASI score [182]. FCO_2_ laser combined with NB‐UVB reported excellent and good repigmentation in 25% and 6.3% [188].

#### 4.6.2 Combination With Laser

308‐nm excimer laser/0.1% tacrolimus showed complete and excellent repigmentation in 35.6% and 42.2% of patients, respectively [166]. Meanwhile, 308‐nm excimer laser/0.03% tacrolimus achieved complete and excellent repigmentation in 36.2% and 45.7% [174]. Comparing double therapy of 308‐nm excimer laser/tacrolimus versus monotherapy, combination therapy reported excellent repigmentation in 23% compared to 11% in 308‐nm excimer laser monotherapy [113]. Pediatric patients treated with 308‐nm excimer laser/halomethasone showed a 70.39% complete recovery [189]. Triple therapy of 308‐nm excimer laser/tacrolimus/calcipotriene showed complete repigmentation after 26 weeks [167]. Significant repigmentation was observed after using FCO_2_ laser/1% phenytoin cream [180]. In a comparison of the combination of FCO_2_ with either NB‐UVB, tacrolimus, or calcipotriol, FCO_2_/NB‐UVB reported 40% excellent repigmentation compared to 30% and 10% in FCO_2_/tacrolimus and FCO_2_/calcipotriol, respectively [216]. When compared with the combination of 5‐FU/FCO_2_ versus monotherapy, 5‐FU/FCO_2_ showed a 90% repigmentation rate compared to 85% in 5‐FU monotherapy [221]. In a study using Er:YAG/5‐FU, 73.3% of patients experienced poor repigmentation and 10% had good‐to‐excellent repigmentation [244].

#### 4.6.3 Combination With Light

Comparing double therapy of 308‐nm excimer light/0.1% tacrolimus versus monotherapy, the combination therapy reported 9.31% complete repigmentation in the face and trunk compared to 47.8% in 308‐nm excimer light monotherapy. Although the face and trunk achieved complete repigmentation, the extremities and acral area only achieved good repigmentation, which was 47.1% in combination therapy and 11.8% in monotherapy [231]. Excimer light and electrocautery needling combination resulted in a mean repigmentation rate of 50.95% compared to 31.29% and 34.86% for excimer light and electrocautery needling monotherapy, respectively [212].

#### 4.6.4 Combination With Microneedling

Microneedling/5‐FU reported good repigmentation of 30% [203]. Additionally, Abdou et al. (2022) reported that microneedling/5‐FU reported excellent and poor repigmentation in 40% and 13.3%, respectively [202]. In comparison with microneedling monotherapy, microneedling/5‐FU reported excellent repigmentation of 48.6% compared to 16.9% [194]. Similar to Chhabra et al. (2021), microneedling combined with 5‐FU showed significant improvement compared with microneedling monotherapy [200]. In another comparison with 5‐FU monotherapy, microneedling/5‐FU reported excellent repigmentation of 47% compared to 4.3% [201]. Initiation of repigmentation was earlier in microneedling/5‐FU (65%) compared to 5‐FU monotherapy (38.7%). Comparing microneedling with either 5‐FU or tacrolimus, microneedling/5‐FU reported good‐to‐excellent repigmentation in 76.7% compared to 63.7% in microneedling/tacrolimus [215]. Nofal et al. (2022) reported that microneedling/TCA had significant repigmentation compared to microneedling/5‐FU or pimecrolimus [206]. Microneedling/pimecrolimus reported 6.7% excellent repigmentation at 6 months follow‐up [228].

#### 4.6.5 Combination of Oral Therapy

Combination of MTX/OMP dexamethasone reported 71.4% perilesional repigmentation and 42.9% intralesional repigmentation compared to 42.9% perilesional repigmentation and 14.3% intralesional repigmentation in OMP dexamethasone monotherapy [208].

#### 4.6.6 Combination of Topical Applications

Topical corticosteroid combined with topical calcineurin inhibitors reported a very good repigmentation rate of 64.5% [190].

#### 4.6.7 Adjuvant to Cell Therapy

Autologous micrografts/NB‐UVB showed a 64.6% repigmentation rate [172]. According to Elgarhy et al. (2020), NCEG homogenized with plasma gel combined with NB‐UVB phototherapy demonstrated a 65% efficiency [179]. Excellent repigmentation was observed after autologous mini‐graft transplantation/NB‐UVB [186]. The mean repigmentation rate was 70.5% in HTIC/NB‐UVB compared to 16.5% in NB‐UVB monotherapy [199]. FUE/CBD and FUE/NB‐UVB had comparable repigmentation rates at the end of 4 months [223]. Significant improvement of the VESTA score after treatment with cultured epidermal autografts/CO_2_ laser was observed [177]. Nineteen and fifteen foci from skin autografts/PUVA reported complete and excellent repigmentation [192]. In addition, significant repigmentation was reported in MKT/motorized MPG [164]. In a comparison of the combination of MPG/microneedling versus monotherapy, MPG/microneedling reported a median repigmentation rate of 49.6% compared to 38.5% and 33.4% in MPG and microneedling monotherapy, respectively [210]. After receiving MKCS/microneedling treatment for 6 months, melanin was significantly increased [162].

Triple therapy of monocyte‐rich PRP/1927 nm fraxel laser/308‐nm excimer laser reported 59% repigmentation [163]. In PRP/308‐nm excimer laser, 40% excellent repigmentation was observed [240]. Using PRP in conjunction with excimer laser, 60% and 13% of the perifollicular and marginal repigmentation, respectively, were described [175]. When compared to FCO_2_ monotherapy, FCO_2_/PRP revealed a significant reduction in VASI score. Tacrolimus monotherapy only achieved good‐to‐excellent repigmentation in 6.8% of cases, compared to 22.5% in combination therapy of bFGF/0.1% tacrolimus [229].

#### 4.6.8 Psychological

Camouflage combined with psychotherapy reported a significant satisfactory response [173].

### 4.7 Triple Combination Therapy

#### 4.7.1 Combination With Laser

Donghaim et al. (2020) found excellent and good repigmentation of 30% and 37.5% following a triple therapy of Er:YAG laser ablation, 5‐FU, and NB‐UVB [187]. Compared to 3% in excimer light monotherapy, 18.2% receiving triple therapy of excimer light, 5‐FU, and microneedling experienced good‐to‐excellent repigmentation [211]. In comparison with FCO_2_/NB‐UVB, FCO_2_/NB‐UVB/0.01% bimatoprost observed a greater rate of repigmentation [224].

#### 4.7.2 Combination With Dermabrasion

Triple therapy of dinoprostone, tacrolimus, and dermabrasion reported good response [245].

#### 4.7.3 Combination With Phototherapy

Good repigmentation was observed after triple therapy of NB‐UVB, vitamin A, and vitamin E [183]. The median rate of repigmentation after triple therapy of NB‐UVB, microneedling, and latanoprost was 47.50% as opposed to 12.50% after NB‐UVB/microneedling double therapy [209].

#### 4.7.4 Combination With Microneedling

At week 52 follow‐up, microneedling/5‐FU/oral corticosteroid complete repigmentation with no recurrence [181]. Compared to 40% in NCES monotherapy, NCES/microneedling/5‐FU reported excellent repigmentation of 84% [218]. In comparison with 32% in microneedling/tacrolimus double therapy, triple therapy of microneedling/calcipotriol/betamethasone reported 60% excellent repigmentation. The most resistant sites, such as the elbows, knees, extremities, and acral areas responded well to the treatment [233].

## 5 New Approach

New approach is studies which did in silico, in vitro, in vivo, or ex vivo experiments for future management of vitiligo. In silico experiments were done using network pharmacology and molecular docking method. Examples of in vitro investigations done include MTT assay for viability analysis, scratch assay for migration studies, transmission electron microscopy for observation of melanosome formation and maturation, western blot analysis, metabolite identification using LC‐QTOF, and prediction of absorption, distribution, metabolism, and excretion using SwissADME web server. While examples of in vivo and ex vivo investigations done for anti‐vitiligo analysis include gross appearance and measurement of the lesioned area, quantitative real‐time RT‐PCR, cytokine assay using ELISA, immunofluorescence to check the infiltration of CD8+ T cells, and DOPA staining to observe the number of melanocytes. Table 3 shows the proposed new approach for management of vitiligo.

**TABLE 3:** Proposed new approach for management of vitiligo.

New approach	Methodology	Conclusion	References
*Drugs*			
Tanshinone IIA	In vivo	Downregulated the Pdk1‐Akt pathway in CD8+ T cells and accumulation of PMEL CD8+ T cells in a mouse model	[246]
Folic acid (FA)	In vitro	Protection of melanocytes from oxidative injury by reducing intracellular ROS levels and upregulating HO‐1 and SOD2. Activation of Nrf2 lead to increased antioxidant proteins. Reduction of HMGB1 lead to inhibition of oxidative stress‐triggered inflammation	[247]
Lenalidomide	In vitro	Reduced IFN‐γ, TNF‐α, IL‐1β, and IL‐6 and increased IL‐4 and IL‐10 levels	[248]
Estradiol	In vitro	An antioxidant protecting melanocytes from oxidative stress	[249]
Methylcobalamin (MeCbl)	In vitro	Attenuated the H_2_O_2_‐induced oxidative stress by activating the Nrf2/HO‐1 pathway	[250]
Tranilast	In vitro	Attenuated the keratinocyte‐derived IL‐1→ under oxidative stress	[251]
Astragaloside IV (AIV)	In vivo	Upregulated TRP‐1, TRP‐2, and MART‐1 expression, indicating that AIV can efficiently induce melanocytes differentiation from bone marrow mesenchymal stem cells	[252]
Flumequine	In vitro and in vivo	Induced increase of melanin content in B16F10 cells and zebrafish larvae by activating p38 MAPK and JNK	[253]
*Herbal*			
*L. shawii* methalonic extract	In vitro and in silico	Increased melanocyte proliferation and migration, enhanced of melanosome formation and maturation, and upregulated MITF, tyrosinase, TRP‐1, and TRP‐2 expression	[254]
*B. gaudichaudii*	In vivo and ex vivo [255], in vitro [256]	Demonstrated high‐permeability and melanogenic properties [255]. *B. gaudichaudii* was less cytotoxic to the B16F10 cells than pure active ingredients, psoralen, and 5‐methoxypsoralen in equivalent doses. Melanin synthesis was enhanced by 300% with the extract compared with the 130% increase with psoralen and 5‐methoxypsoralen [256]	[255], [256]
Paeoniflorin	In vivo [257], in vitro [258]	Increased cell proliferation and melanin biosynthesis via activating CREB and ERK [257]. Resisted H_2_O_2_‐induced oxidative stress by activating *JNK/Nrf2/HO‐1* signaling [258]	[257], [258]
Artiri La Li Honey Pill (ALLHP)	In vivo	Increased melanin‐containing hair follicles and the epidermal melanin content, repaired the skin cell morphology, increased the tyrosine content in serum and skin, and reduced the MDA content	[259]
Furocoumarin	In vitro [260, 261, 262], in vivo [260]	Promising candidate as anti‐vitiligo [262]. Amine derivatives of furocoumarin, 5‐(morpholinomethyl)‐3‐phenyl‐7*H*‐furo[3,2‐*g*]chromen‐7‐one promoted melanin synthesis by stimulating the nuclear translocation of β‐catenin, which activated MITF transcription [261]. Furocoumarin derivative, 5‐((diethylamino) me‐13 thyl)‐3‐phenyl‐7H‐furo [3, 2‐g] chromen‐7‐one attenuated the depigmentation of the C57BL/6 vitiligo mice model by increasing the numbers of melanin‐containing hair follicles, melanogenic protein, and melanogenesis‐relative genes expression in skin tissues [260]	[262], [261], [260]
*Sorbus commixta* twig ethanol extract	In vitro	Potential therapeutic activity in vitiligo	[263]
Caraway tablet (CWT)	In vivo	Promoted tyrosinase activity and melanin synthesis in B16 cells via activation of the p38 MAPK and PKA signaling pathways. CWT attenuated the detrimental changes induced by hydroquinone or hydrogen peroxide in animal models of vitiligo by stimulation of pigmentation and reestablishment of redox balance, blocking melanocyte cell death from damage. CWT exhibited an equivalent effect to Baidianfeng capsule (BC)	[264]
*Psoralea corylifolia* (babchi) seeds	In vitro	Enhanced the proliferation of melanocytes	[265]
Vitexin	In vitro	Vitexin protected melanocytes from oxidative stress by activating MAPK‐Nrf2/ARE signaling pathway	[266]
*Lycium barbarum* polyaccharide	In vitro	Demonstrated anti‐vitiligo activity	[267]
Diterpenes from Euphoria antiquorum L. Fitoterapia	In vitro	Exhibited better melanin synthesis than 8‐MOP. The activity of ingenol diterpenoid 12 (203.1%) were nearly 2‐fold stronger than 8‐MOP (124.38%) on melanin synthesis in murine B16 cells	[268]
*Lespedeza bicolor* extract	In vitro and in vivo	Demonstrated anti‐vitiligo activity	[269]
Apigenin	In vitro	Apigenin enhanced cell viability under H_2_O_2_. Apigenin increased SOD, CAT, and GSH‐Px activities and inhibited MDA levels	[270]
Ginsenoside Rk1	In vitro	Ginsenoside Rk1 protected melanocytes from H_2_O_2_‐induced oxidative stress by regulating Nrf2/HO‐1 protein expression	[271]
6‐Shogaol (6‐SG)	In vitro	6‐Shogaol protected melanocytes against oxidative stress by the activation of the Nrf2‐antioxidant response element signaling pathway	[272]
Glycyrrhizin (GR)	In vitro	GR protected human melanocytes from H_2_O_2_‐induced oxidative damage via the Nrf2‐dependent induction of HO‐1	[273]
Paeonol	In vitro	Paeonol protected melanocytes against H_2_O_2_‐induced oxidative stress by Nrf2 mediated antioxidant pathways	[274]
Natural citrus flavanone 5‐demethylnobiletin	In vitro	5‐demethylnobiletin demonstrated anti‐vitiligo activity	[275]
Epimedium brevicornum Maxim extract	In vitro and in vivo	Epimedium brevicornum Maxim demonstrated pigmenting activity	[276]
Pinostrobin	In vitro	Pinostrobin demonstrated anti‐vitiligo activity	[277]
*Cirsium japonicum* flower extract	In vitro	*C. japonicum* flower extract (CFE) stimulated melanogenesis through cAMP signaling	[278]
*Vernonia anthelmintica* (L.) Willd	In vitro and in vivo	*Vernonia anthelmintica (L.) Willd*. enhanced melanogenesis	[279]
*Ginkgo biloba* extract	In vitro	*Ginkgo biloba* extract EGb761 protected melanocytes from H_2_O_2_‐induced oxidative stress by activating Nrf2	[280]
Ultradeformable liposomes (UDL) of psoralen derivatives	In vitro	5‐methoxypsoralen (5‐MOP) UDL followed by 8‐methoxypsoralen (8‐MOP) UDL revealed superior efficacy over psoralen UDL. Efficacy of psoralen derivatives at low dose can address the tolerability and safety issues in future	[281]
Berberine	In vitro	Berberine protected melanocytes against oxidative stress via its antioxidative activity and inhibition of NFκB	[282]
Maclurin	In vitro	Maclurin demonstrated anti‐vitiligo activity	[283]
Thymoquinone	In vitro	Thymoquinone enhanced melanogenesis	[284]
Ethalonic extract of *Melia azedarach* L.	In vitro	Ethalonic extract of *Melia azedarach* L. induced melanogenesis by upregulating the MITF gene through the cAMP‐PKA‐CREB signaling pathway	[285]
Puerarin	In vitro and in vivo	Puerarin demonstrated anti‐vitiligo activity	[286]
Naringenin	In silico	Naringenin enhanced melanogenesis and has good pharmacokinetics	[287]
Bailing tablet	In silico	Bailing tablet demonstrated anti‐vitiligo activity	[288]
*Scaffold*			
HGDexMA hydrogel	In vivo	Vitiligo mice treated with HGDexMA/tofacitinib/α‐MSHMNs showed faster pigmentation speed and were able to achieve complete pigmentation of the depigmented area	[289]
Berberine‐loaded hyalurosomes	In vitro, in vivo, and ex vivo	Berberine‐loaded hyalurosomes showed promising skin permeation and deposition properties with antioxidant and anti‐inflammatory effects. This delivery system utilized a new platform for the localized topical treatment of vitiligo to minimize the side effects of systemic therapies	[290]
*Compound*			
Melanosomes	In vivo	Melanosomes showed potential as a universal platform for the self‐supply of melanin by self‐driven melanin synthesis with exogenous supplementation	[291]
H‐2	In vitro	H‐2 alleviated oxidative stress damage in *C. elegans* and B16‐F10, suppressed antioxidant defenses and transcription factors DAF‐16/FOXO	[292]
PEGylated catalase	In vitro	PEGylated catalase demonstrated anti‐vitiligo activity	[293]
MicroRNA‐145‐5p	In vitro	MicroRNA‐145‐5p protected human melanocytes against oxidative damage by targeting transient receptor potential melastatin 2	[294]
MicroRNA‐637	In vitro	MicroRNA‐637 relieved oxidative stress‐stimulated melanocyte injury via downregulating TRPM2 expression	[295]
4‐octyl itaconate (4‐OI)	In vitro and in vivo	4‐OI protected melanocytes and keratinocytes from UVB‐induced apoptosis by Nrf2 activation‐dependent ROS inhibition	[296]
*Phototherapy*			
Lower irradiation dose of 308‐nm monochromatic excimer light	In vitro and in vivo	Lower irradiation dose of 308 nm monochromatic excimer light was sufficient and more suitable for repigmentation	[297]
*Cell‐based*			
Direct reprogramming of fibroblasts into melanocytes	In vivo	Lentivirus packaging system protocol was presented to produce transcription factors selected for reprogramming skin cells to melanocytes, including Sox10, Mitf, Pax3, Sox2, Sox9, and Snai2. Direct reprogramming of fibroblasts to melanocytes could be a successful new therapeutic strategy for vitiligo	[298]
Mouse bone marrow mesenchymal stem cells into melanocytes (MiMels)	In vitro and in vivo	MiMels had a typical melanocyte morphology and same biological functions as normal melanocytes. MiMels was successfully applied in mouse tissue‐engineered experiments	[299]
Extracellular fraction of adipose tissue	In vitro	Adipose tissue extracellular fraction demonstrated anti‐vitiligo activity	[300]
iPSC‐derived melanocytes	In vitro and n‐vivo	iPSCs of vitiligo patients have the potential to differentiate into melanocytes, with functionality observed both in vitro and in vivo. iPSC‐derived melanocytes possessed capacities of hair follicle and epidermis reconstitution and long‐term function maintenance	[301]
*Others*			
Serum from healthy individuals	In vitro	Serum treatment following moxibustion at the “Jiudianfeng” point promoted melanocyte proliferation and melanin synthesis	[302]
*Combination*			
*Buddleja officinalis* combined with blue light‐emitting diode	In vitro	Combined therapies of *Buddleja officinalis* (BO) and blue light irradiation demonstrated anti‐vitiligo activity	[303]
NB‐UVB phototherapy combined with adipose‐derived stem cells	In vivo	Combined NB‐UVB/adipose‐derived stem cells demonstrated anti‐vitiligo activity	[304]
Aminoguanidine combined with NB‐UVB phototherapy	In vivo	Significant repigmentation of vitiligous lesions treated with iNOS inhibitor aminoguanidine/NB‐UVB therapy	[305]
Hesperidin combined with trimethylpsoralen	In vivo	Hesperidin/trimethylpsoralen combination acted as an effective phytochemotherapy agent	[306]
Baicalin or berberine ultradeformable vesicles or their combination	In vitro	Baicalin or berberine ultradeformable vesicles or their combination demonstrated anti‐vitiligo activity	[307]

### 5.1 Prescription Medications

Anti‐vitiligo analysis using vitiligo animal models was investigated with tanshinone IIA, flumequine, lenalidomide, and astragaloside IV. Tanshinone IIA alleviated vitiligo by downregulating the Pdk1‐Akt pathway in CD8+ T cells in vitiligo mice models [246]. Flumequine induced an increase in melanin content in zebrafish larvae by activating p38 MAPK and JNK [253]. Lenalidomide reduced the level of proinflammatory cytokines IFN‐γ, TNF‐α, IL‐1β, and IL‐6 and increased IL‐4 and IL‐10, preventing depigmentation in vitiligo mice models [248]. Astragaloside IV increased TRP‐1, TRP‐2, and MART‐1 expression, inducing melanocytes' differentiation from bone marrow mesenchymal stem cells [252].

In vitro anti‐vitiligo activity was investigated with folic acid, estradiol, methylcobalamin, flumequine, and tranilast. Folic acid's role in vitiligo was explored by Du et al. (2021) [247]. Folic acid was found to protect melanocytes from oxidative injury by reducing intracellular ROS levels and increasing antioxidant enzymes, such as HO‐1 and SOD2. Furthermore, folic acid activated Nrf2, leading to increased expression of antioxidants. Folic acid also reduced HMGB1 levels in melanocytes, inhibiting oxidative stress‐triggered inflammation [247]. Local estradiol functions as an antioxidant, protecting melanocytes from oxidative stress [249]. Methylcobalamin reduced H_2_O_2_‐induced oxidative stress by activating the Nrf2/HO‐1 pathway [250]. Additionally, flumequine induced an increase in melanin content in B16F10 cells by activating p38 MAPK and JNK [253]. Tranilast reduced IL‐1β induced oxidative stress [251].

### 5.2 Herbal Extracts

Anti‐vitiligo analysis of herbal extracts using vitiligo animal models was investigated. *B. gaudichaudii* possesses melanogenic properties [255]. Paenoflorin administration to vitiligo mice models exhibited therapeutic effects [257]. Oral administration of furocoumarin by C57BL/6 vitiligo mice models reduced the depigmentation process and increased the numbers of melanin‐containing hair follicles, melanogenic protein, and melanogenesis‐relative genes expression [260]. Additionally, hesperidin/trimethylpsoralen [306] and puerarin [286] had therapeutic potential in vitiligo. ALLH increased the number of melanin‐containing hair follicles and the epidermal melanin content in the skin of experimental vitiligo animals, increased tyrosine level, and reduced the MDA content [259]. Caraway therapy decreased the detrimental effects induced by H_2_O_2_ by reestablishing redox balance and blocking damage to melanocytes. Furthermore, *Lespedeza bicolor* extract [269] and *Vernonia anthelmintica* (L.) Willd. [279] had therapeutic potential against vitiligo. Caraway exhibited an equivalent effect to Baidianfeng capsule [264].

In vitro anti‐vitiligo activity of herbal extracts was investigated. L‐shawii methalonic extract increased melanocyte proliferation and migration, enhanced melanosome formation and maturation, and increased melanogenesis‐related proteins including MITF, tyrosinase, TRP‐1, and TRP‐2 [254]. In vitro experiments with *B. gaudichaudii* exhibited 300% enhancement in melanogenesis [256]. Amine derivatives of furocoumarin promoted melanogenesis by stimulating the nuclear translocation of β‐catenin, which activates MITF transcription [261]. Ethanolic extract of *Melia azedarach* L. induced melanogenesis by increasing MITF gene through cAMP‐PKA‐CERB signaling pathway [285]. Similarly, *cirsium japonicum* flower extract induced melanogenesis through cAMP signaling [278]. Caraway increased tyrosinase activity and melanogenesis in B16 cells via activation of the p38 MAPK and PKA signaling pathways. Paeoniflorin increased melanogenesis by activating CERB and ERK at a concentration of 10 μg/mL [257]. In addition, paeoniflorin also inhibited H_2_O_2_‐induced oxidative stress by activating JNK/Nrf2/HO‐1 signaling [258].

Psoralen derivatives, 5‐MOP and 8‐MOP in the form of ultradeformable vesicles revealed better efficacy than psoralen as the promising approach for vitiligo [281]. However, *E. antiquorum* L. exhibited significantly better melanogenesis in vitro than 8‐MOP in murine B16 cells. Especially, compound ingenol diterpenoid 12 was specific against vitiligo [268]. Ginsenoside Rk1, a major compound isolated from ginseng, protected melanocytes from H_2_O_2_‐induced oxidative stress by regulating Nrf2/HO‐1 protein expression [271]. Similarly, *Ginkgo biloba* extract EGb761 [280], glycyrrhizin [273], 6‐shogaol [272], and paeonol [274] protected melanocytes against H_2_O_2_‐induced oxidative stress by activating the Nrf‐2 signaling pathway. Berberine protected melanocytes against H_2_O_2_‐induced oxidative stress by its antioxidative activity [282]. In addition, baicalin or berberine ultradeformable vesicles or their combinations represented promising nanosytem‐based adjuvants for the treatment of vitiligo [307]. Similarly, apigenin increased SOD, CAT, and GSH‐Px activities and inhibited MDA levels via regulation of Nrf2 [270]. Vitexin inhibited oxidative stress by activating the MAPK‐Nrf2/ARE signaling pathway [266]. Another herbal extract proven to have therapeutic potential in vitiligo includes ethanol extract of *S. commixta* twigs [263], *P. coryfolia* seeds [265], *L. barbarum* polysaccharide [267], Epimedium brevicornum Maxim extract [276], *Vernonia anthelmintica* (L.) Willd [279], thymoquinone [284], natural citrus flavanone 5‐demethylnobiletin [275], pinostrobin [277], maclurin [283], and puerarin [286]. Naringenin and bailing tablet were reported to induce melanogenesis in silico [287, 288].

### 5.3 Scaffold

Vitiligo mice treated with HGDexMA/tofacitinib/α‐MSHMNs hydrogel showed an increased repigmentation rate and achieved complete repigmentation [289]. Berberine‐loaded hyalurosomes showed promising skin permeation with antioxidant and anti‐inflammatory effects in in vitro, in vivo, and ex vivo experiments [290].

### 5.4 Compound

An in vivo study by Sun et al. (2021) demonstrated that melanosomes had the potential for universal self‐supply of melanin through self‐melanogenesis stimulated by exogenous supplementation [291]. An in vivo and in vitro study by Xie et al. (2022) reported that 4‐OI protected melanocytes and keratinocytes against UVB‐induced apoptosis by activating the Nrf2 signaling pathway [296]. According to an in vitro study by Song et al. (2023), H‐2 can reduce oxidative injury in *C. elegans* and B16‐F10 cells [292]. An in vitro study by Huang et al. (2021) reported that microRNA‐145‐5p protected melanocytes against oxidative injury by targeting transient receptor potential melastatin 2 [294]. Furthermore, according to an in vitro study by Sun et al. (2022), microRNA‐637 protected melanocytes against oxidative injury by reducing TRPM2 expression [295]. It was discovered that PEGylated catalase had therapeutic potential against vitiligo [293].

### 5.5 Phototherapy

Lower irradiation dose of 308‐nm monochromatic excimer light was found to have repigmentation potential in vitiligo in both in vitro and in vivo studies [297].

### 5.6 Cell‐Based

Direct reprogramming of fibroblasts to melanocytes by utilizing transcription factors including SOX2, SOX9, SOX10, MITF, PAX3, and SNAI1 could be a successful new therapeutic strategy for vitiligo [298]. In addition, adipose tissue extracellular fraction could be a useful natural approach to treat vitiligo [300]. Another idea is mouse bone marrow mesenchymal stem cells, which have a typical melanocyte morphology and the same biological functions as human melanocytes [299]. Furthermore, iPSCs of vitiligo patients can differentiate into melanocytes and possess capacities of reconstituting hair follicles and epidermis with long‐term functionality, as observed in both in vitro and in vivo studies [301]. Additionally, serum from healthy individuals was proven to promote melanogenesis in an in vitro study [302].

### 5.7 Combination Therapy

Combined therapies of *Buddleja officinalis* and blue light irradiation were found to have repigmentation potential in vitiligo in an in vitro study [303]. While in vivo studies by Bian et al. (2022) and Mansourpour et al. (2019) revealed that combination therapies of adipose‐derived stem cells/NB‐UVB [304] and aminoguanidine/NB‐UVB [305] had repigmentation potential in vitiligo.

## 6 Conclusions

Vitiligo is a disease with a complex and multifactorial pathogenesis according to the characteristics of the patients and has a great impact on the quality of life. Morphology and environmental alteration, oxidative stress, and autoimmunity play major roles in the development of vitiligo. Furthermore, multiple affected family members have a higher risk compared to single cases. Current accessible therapies include oral medications, topical ointments, phototherapy, injections, grafts, combination therapies, and psychotherapies. Patients should be informed on therapeutic options available to treat their conditions. Unfortunately, not all patients respond to the same treatments. The treatment and management of vitiligo continue to pose a significant challenge for both researchers and dermatologists. The success rate for vitiligo treatments varies according to the type, age, ethnicity, and duration of vitiligo. Overall, the research suggests that implementing early intervention can enhance the repigmentation and inhibit disease progression, especially in patients with progressive type.

Emerging treatments for vitiligo are moving beyond conventional immunosuppression toward more precise and targeted approaches. Among the most notable advancements are JAK inhibitors, such as topical ruxolitinib, which effectively block IFN‐γ‐mediated JAK–STAT pathway involved in melanocyte destruction. In parallel, active compound incorporated bioscaffolds such as HGDexMA/tofacitinib/α‐MSHMNs incorporated hydrogel have been developed as an alternative for vitiligo treatment. The combination showed a fast pigmentation rate and is able to achieve complete pigmentation. Regenerative strategies focusing on melanocyte stem cell biology and the restoration of the epidermal microenvironment are gaining traction. As such, direct reprogramming of fibroblasts to melanocytes by utilizing transcription factors including SOX2, SOX9, SOX10, MITF, PAX3, and SNAI1 could be a successful new therapeutic strategy for vitiligo. Furthermore, future treatment approaches may aim to restore skin homeostasis rather than merely suppressing inflammation, potentially leading to disease reversal. These approaches aim to reestablish skin homeostasis and promote long‐lasting repigmentation, potentially leading to more durable disease control. Although still largely in the experimental phase, they represent a promising frontier in vitiligo therapy. Together, these emerging modalities point toward a more personalized and mechanistically driven future in vitiligo management.

## Author Contributions

Conceptualization, N.S., M.M.; writing and original draft preparation, N.S., M.M.; writing, review, and editing, N.S., N.I.M.F., M.B.F., and M.M.; supervision, M.B.F., M.M.; project administration, M.M.; funding acquisition, M.M. All authors have read and agreed to the published version of the manuscript.

## Ethics Statement

The authors have nothing to report.

## Consent

The authors have nothing to report.

## Conflicts of Interest

The authors declare no conflicts of interest.

## Data Availability

Data sharing not applicable to this article as no datasets were generated or analyzed during the current study.
